# Supplementary material for: Body Image Satisfaction, Food Consumption, Diet Quality, and Emotional Management in Adolescence: A Longitudinal Analysis from the SI! Program for Secondary Schools Trial
Source: Nutrients. 2025 Dec 12;17(24):3882. doi: 10.3390/nu17243882 (PMC12736312; doi:10.3390/nu17243882)
Supplement: Supplementary file 1 [file nutrients-17-03882-s001.zip › 2025.12.05_Supplemental material_BIS_Nutrients_clean.pdf]

Supplementary Material

Supplementary Tables 2

Supplemental Table S1. 2

Supplemental Table S2. 7

Supplemental Table S3. 9

Supplemental Table S4. 10

Supplemental Table S5. 13

Supplemental Table S6 16

Supplemental Table S7. 17

Supplemental Table S8. 18

Supplemental Table S9. 20

Supplemental Table S10. 22

Supplementary Figures 23

Supplemental Figure S1 23

Supplemental Figure S2 25

Supplemental Figure S3 26

Supplementary Tables

Supplemental Table S1. The STrengthening the Reporting of OBservational studies in Epidemiology (STROBE)-nut: An extension of the STROBE statement for nutritional epidemiology.

Item	Item nr	STROBE recommendations	Extension for Nutritional Epidemiology studies (STROBE-nut)	Reported on page #
**Title and abstract**	1	(a) Indicate the study's design with a commonly used term in the title or the abstract.<br>(b) Provide in the abstract an informative and balanced summary of what was done and what was found.	**nut-1** State the dietary/nutritional assessment method(s) used in the title, abstract, or keywords.	1, 3, 4
**Introduction**				
Background rationale	2	Explain the scientific background and rationale for the investigation being reported.		5
Objectives	3	State specific objectives, including any pre-specified hypotheses.		5
**Methods**				
Study design	4	Present key elements of study design early in the paper.		5, 6
Settings	5	Describe the setting, locations, and relevant dates, including periods of recruitment, exposure, follow-up, and data collection.	**nut-5** Describe any characteristics of the study settings that might affect the dietary intake or nutritional status of the participants, if applicable.	5-7
Participants	6	a) Cohort study—Give the eligibility criteria, and the sources and methods of selection of participants. Describe methods of follow-up.<br>Case-control study—Give the eligibility criteria, and the sources and methods of case ascertainment and control selection. Give the rationale for the choice of cases and controls.	**nut-6** Report particular dietary, physiological or nutritional characteristics that were considered when selecting the target population.	5-7

Item	Item nr	STROBE recommendations	Extension for Nutritional Epidemiology studies (STROBE-nut)	Reported on page #
		Cross-sectional study—Give the eligibility criteria, and the sources and methods of selection of participants.<br>(b) Cohort study—For matched studies, give matching criteria and number of exposed and unexposed.<br>Case-control study—For matched studies, give matching criteria and the number of controls per case.		
Variables	7	Clearly define all outcomes, exposures, predictors, potential confounders, and effect modifiers. Give diagnostic criteria, if applicable.	**nut-7.1** Clearly define foods, food groups, nutrients, or other food components.<br>**nut-7.2** When using dietary patterns or indices, describe the methods to obtain them and their nutritional properties.	6-9, Supp Table 2.
Data sources - measurements	8	For each variable of interest, give sources of data and details of methods of assessment (measurement). Describe comparability of assessment methods if there is more than one group.	**nut-8.1** Describe the dietary assessment method(s), e.g., portion size estimation, number of days and items recorded, how it was developed and administered, and how quality was assured. Report if and how supplement intake was assessed.<br>**nut-8.2** Describe and justify food composition data used. Explain the procedure to match food composition with consumption data. Describe the use of conversion factors, if applicable.<br>**nut-8.3** Describe the nutrient requirements, recommendations, or dietary guidelines and the evaluation approach used to compare intake with the dietary reference values, if applicable.<br>**nut-8.4** When using nutritional biomarkers, additionally use the STROBE Extension for Molecular Epidemiology	6-9

Item	Item nr	STROBE recommendations	Extension for Nutritional Epidemiology studies (STROBE-nut)	Reported on page #
			(STROBE-ME). Report the type of biomarkers used and their usefulness as dietary exposure markers.<br>**nut-8.5** Describe the assessment of nondietary data (e.g., nutritional status and influencing factors) and timing of the assessment of these variables in relation to dietary assessment.<br>**nut-8.6** Report on the validity of the dietary or nutritional assessment methods and any internal or external validation used in the study, if applicable.	
Bias	9	Describe any efforts to address potential sources of bias.	**nut-9** Report how bias in dietary or nutritional assessment was addressed, e.g., misreporting, changes in habits as a result of being measured, or data imputation from other sources	6-9, Supp Table 10
Study Size	10	Explain how the study size was arrived at.		5-6
Quantitative variables	11	Explain how quantitative variables were handled in the analyses. If applicable, describe which groupings were chosen and why.	**nut-11** Explain categorization of dietary/nutritional data (e.g., use of N-tiles and handling of nonconsumers) and the choice of reference category, if applicable.	6-9
Statistical Methods	12	(a) Describe all statistical methods, including those used to control for confounding<br>(b) Describe any methods used to examine subgroups and interactions.<br>(c) Explain how missing data were addressed.<br>(d) Cohort study—If applicable, explain how loss to follow-up was addressed.<br>Case-control study—If applicable, explain how matching of cases and controls was addressed.<br>Cross-sectional study—If applicable, describe analytical methods taking account of sampling strategy.<br>(e) Describe any sensitivity analyses.	**nut-12.1** Describe any statistical method used to combine dietary or nutritional data, if applicable.<br>**nut-12.2** Describe and justify the method for energy adjustments, intake modeling, and use of weighting factors, if applicable.<br>**nut-12.3** Report any adjustments for measurement error, i.e,. from a validity or calibration study.	6-10

Item	Item nr	STROBE recommendations	Extension for Nutritional Epidemiology studies (STROBE-nut)	Reported on page #
**Results**				
Participants	13	(a) Report the numbers of individuals at each stage of the study—e.g., numbers potentially eligible, examined for eligibility, confirmed eligible, included in the study, completing follow-up, and analyzed.<br>(b) Give reasons for non-participation at each stage.<br>(c) Consider use of a flow diagram.	**nut-13** Report the number of individuals excluded based on missing, incomplete or implausible dietary/nutritional data.	5-10
Descriptive data	14	(a) Give characteristics of study participants (e.g., demographic, clinical, social) and information on exposures and potential confounders<br>(b) Indicate the number of participants with missing data for each variable of interest<br>(c) Cohort study—Summarize follow-up time (e.g., average and total amount)	**nut-14** Give the distribution of participant characteristics across the exposure variables if applicable. Specify if food consumption of total population or consumers only were used to obtain results.	10, Table 1
Outcome data	15	Cohort study—Report numbers of outcome events or summary measures over time.<br>Case-control study—Report numbers in each exposure category, or summary measures of exposure.<br>Cross-sectional study—Report numbers of outcome events or summary measures.		10-13, Figures 1-3, Tables 2-3, Supp Tables 3-9, Supp Figure 1-3
Main results	16	(a) Give unadjusted estimates and, if applicable, confounder-adjusted estimates and their precision (e.g., 95% confidence interval).<br>Make clear which confounders were adjusted for and why they were included.<br>(b) Report category boundaries when continuous variables were categorized.	**nut-16** Specify if nutrient intakes are reported with or without inclusion of dietary supplement intake, if applicable.	10-13, Figures 1-3, Tables 2-3, Supp Tables 3-9

Item	Item nr	STROBE recommendations	Extension for Nutritional Epidemiology studies (STROBE-nut)	Reported on page #
		(c) If relevant, consider translating estimates of relative risk into absolute risk for a meaningful time period.		
Other analyses	17	Report other analyses done—e.g., analyses of subgroups and interactions and sensitivity analyses.	**nut-17** Report any sensitivity analysis (e.g., exclusion of misreporters or outliers) and data imputation, if applicable.	Supp Table 10
**Discussion**				
Key results	18	Summarize key results with reference to study objectives.		13-16
Limitation	19	Discuss limitations of the study, taking into account sources of potential bias or imprecision. Discuss both direction and magnitude of any potential bias.	**nut-19** Describe the main limitations of the data sources and assessment methods used and implications for the interpretation of the findings.	16-17
Interpretation	20	Give a cautious overall interpretation of results considering objectives, limitations, multiplicity of analyses, results from similar studies, and other relevant evidence.	**nut-20** Report the nutritional relevance of the findings, given the complexity of diet or nutrition as an exposure.	13-16
Generalizability	21	Discuss the generalizability (external validity) of the study results.		13-17
**Other information**				
Funding	22	Give the source of funding and the role of the funders for the present study and, if applicable, for the original study on which the present article is based.		119
Ethics			**nut-22.1** Describe the procedure for consent and study approval from ethics committee(s).	5, 18
Supplementary material			**nut-22.2** Provide data collection tools and data as online material or explain how they can be accessed.	28, Supp Tables 1-10e

Supplemental Table S2. Classification of food items in relation to diet quality.

FOOD GROUPS	PREFERABLE	INTERMEDIATE	LOW
**Recommended foods**			
Water	- Water	- Diet drinks*	- Sweetened drinks*
Cereals	- Porridge, oat meal, cereals, muesli, unsweetened<br>- Wholemeal bread, dark roll, dark crispbread	- Breakfast cereals, muesli, sweetened<br>- White bread, white roll, white crispbread<br>- Pasta, noodles, rice, cereals, milled	- Biscuits, packaged cakes, pastries, puddings*<br>- Savoury pastries, fritters*
Vegetables and legumes	- Cooked vegetables, potatoes, beans<br>- Raw vegetables<br>- Meat replacement products, plant milk	-	- Fried potatoes, potato croquettes*<br>- Crisps, maize (corn) crisps, popcorn*
Fruits	- Fresh fruits without added sugar	- Fresh fruits with added sugar	- Fruit juices*<br>- Jam*
Nuts	- None	- Nuts, seeds, dried fruits	- None
Dairy products	- Plain unsweetened milk<br>- Plain unsweetened yogurt or kefir	- Sweetened milk<br>- Sweet yogurt, fermented milk beverages<br>- Cheese (sliced and grated cheese)	- Spreadable cheese*<br>- Ice cream, milk or fruit-based bars*
Fish	- Fresh or frozen fish, not fried	- Fried fish, fish fingers	- None
Eggs	- Boiled or poached eggs	- Fried or scrambled eggs	- None
Meat	- Fresh meat, not fried	- Fried meat	- Cold cuts, preserved, ready to cook meat products*<br>- Hamburgers, hot dogs, kebabs, wraps, falafel*
**Non-recommended foods**	- None	- Spreadable fats (butter and margarine low fat*	- Spreadable fats (mayonnaise, butter, margarine)*<br>- Chocolate or nut-based*<br>- Honey*<br>- Ketchup*<br>- Pizza as main dish*<br>- Chocolate, candy bars*<br>- Candies, loose candies, marshmallows*

Adapted from Iglesia I et al 20201 and Vyncke K et al 20132. * These food groups were considered non-recommended foods for the calculation of the Diversity and Equilibrium component of the Diet Quality Index for Adolescents (DQI-A).

[1] Iglesia I, Intemann T, De Miguel-Etayo P, Pala V, Hebestreit A, Wolters M, et al. Dairy Consumption at Snack Meal Occasions and the Overall Quality of Diet during Childhood. Prospective and Cross-Sectional Analyses from the IDEFICS/I.Family Cohort. Nutrients. 2020;12(3).; [2] Vyncke K, Cruz Fernandez E, Fajo-Pascual M, Cuenca-Garcia M, De Keyzer W, Gonzalez-Gross M, et al. Validation of the Diet Quality Index for Adolescents by comparison with biomarkers, nutrient, and food intakes: the HELENA study. Br J Nutr. 2013;109(11):2067-78.

Supplemental Table S3. Distribution of the sample based on body image satisfaction at baseline, 2- and 4-year follow-up.

	**BASELINE** (n=1310)				**2-YEAR FOLLOW-UP** (n=1208)				**4-YEAR FOLLOW-UP** (n=1108)			
	**Overall**	**Boys**	**Girls**	**p-value**	**Overall**	**Boys**	**Girls**	**p-value**	**Overall**	**Boys**	**Girls**	**p-value**
**Body image satisfaction, n (%)**												
Satisfied	437 (33.4%)	169 (25.0%)	268 (42.3%)	**<0.001**	468 (38.7%)	209 (33.1%)	259 (44.9%)	**<0.001**	363 (32.8%)	184 (31.8%)	179 (33.8%)	**<0.001**
Desire to lose weight	629 (48.0%)	348 (51.4%)	281 (44.4%)		517 (42.8%)	270 (42.8%)	247 (42.8%)		496 (44.8%)	217 (37.5%)	279 (52.6%)	
Desire to gain weight	244 (18.6%)	160 (23.6%)	84 (13.3%)		223 (18.5%)	152 (24.1%)	71 (12.3%)		249 (22.5%)	177 (30.6%)	72 (13.6%)	

Values are expressed as frequency (percentage). P-values for gender differences were calculated by chi-square test. Sigificant differences (p≤0.05) are presented in bold

Supplemental Table S4. Food consumption at baseline, 2- and 4-year follow-up by BIS gender.

	BOYS					
	BASELINE (n=672)		2-YEAR FOLLOW-UP (n=618)		4-YEAR FOLLOW-UP (n=562)	
	**mean (95% CI)** †	**OR (95% CI)** ‡	**mean (95% CI)** †	**OR (95% CI)** ‡	**mean (95% CI)** †	**OR (95% CI)** ‡
Fruits and vegetables (serving/day)						
Satisfied	2.4 (2.1; 2.7)	[Ref]	2.1 (1.8; 2.3)	[Ref]	2.1 (1.8; 2.4)	[Ref]
Desire to Lose	2.4 (2.1; 2.6)	0.90 (0.57; 1.41)	2.0 (1.8; 2.3)	1.12 (0.71; 1.76)	2.0 (1.7; 2.3)	1.14 (0.69; 1.90)
Desire to Gain	2.1 (1.8; 2.5)	0.73 (0.44; 1.20)	2.0 (1.7; 2.3)	0.70 (0.42; 1.16)	2.1 (1.8; 2.4)	0.98 (0.59; 1.61)
Fast food (serving/week)						
Satisfied	5.3 (4.1; 6.5)	[Ref]	4.3 (3.7; 5.0)	[Ref]	**3.8 (3.1; 4.4)**[b]	[Ref]
Desire to Lose	5.1 (4.2; 6.0)	0.94 (0.53; 1.66)	**3.6 (2.9; 4.2)**[c]	0.61 (0.31; 1.20)	**3.3 (2.6; 3.9)**[c]	0.85 (0.51; 1.42)
Desire to Gain	5.8 (4.5; 7.1)	1.37 (0.76; 2.49)	**5.2 (4.4; 6.0)**[c]	1.31 (0.70; 2.46)	**5.3 (4.6; 6.0)**[b,c]	**2.14 (1.32; 3.49)**
Sugared and sweetened beverages (serving/week)						
Satisfied	18.2 (15.5; 20.9)	[Ref]	14.2 (12.5; 15.9)	[Ref]	10.3 (8.6; 11.9)	[Ref]
Desire to Lose	15.8 (13.7; 17.9)	0.66 (0.40; 1.07)	11.4 (9.8; 13.1)	**0.61 (0.38; 0.99)**	9.0 (7.3; 10.6)	0.61 (0.36; 1.02)
Desire to Gain	17.9 (14.9; 20.8)	1.22 (0.74; 2.03)	13.3 (11.2; 15.4)	1.01 (0.62; 1.64)	11.6 (9.9; 13.4)	0.95 (0.58; 1.54)
Sweets (serving/week)						
Satisfied	12.4 (10.2; 14.6)	[Ref]	**9.6 (6.7; 12.4)**[b]	[Ref]	7.6 (6.0; 9.2)	[Ref]
Desire to Lose	10.7 (9.0; 12.3)	0.70 (0.43; 1.13)	**9.3 (6.5; 12.1)**[c]	0.85 (0.52; 1.40)	**5.4 (3.8; 7.0)**[c]	**0.48 (0.28; 0.81)**
Desire to Gain	12.7 (10.3; 15.2)	1.19 (0.72; 1.96)	**12.8 (9.7; 15.9)**[b,c]	**2.44 (1.49; 4.0)**	**10.3 (8.6; 12.0)**[c]	1.61 (1.00; 2.57)
Snacks (serving/week)						
Satisfied	2.4 (1.8; 3.0)	[Ref]	2.0 (1.4; 2.6)	[Ref]	1.5 (1.1; 1.9)	[Ref]
Desire to Lose	2.1 (1.6; 2.6)	0.87 (0.47; 1.60)	**1.7 (1.1; 2.3)**[c]	1.00 (0.51; 1.96)	1.4 (1.0; 1.9)	0.97 (0.42; 2.28)
Desire to Gain	2.7 (2.0; 3.3)	1.54 (0.84; 2.80)	**2.6 (1.9; 3.2)**[c]	1.74 (0.93; 3.25)	2.0 (1.6; 2.5)	1.45 (0.69; 3.05)
Nuts (serving/week)						
Satisfied	2.7 (2.0; 3.4)	[Ref]	2.0 (1.4; 2.6)	[Ref]	**1.7 (1.3; 2.2)**[b]	[Ref]
Desire to Lose	2.2 (1.6; 2.7)	0.69 (0.40; 1.19)	2.2 (1.6; 2.7)	1.36 (0.76; 2.43)	**1.5 (1.0; 1.9)**[c]	0.69 (0.35; 1.36)
Desire to Gain	2.6 (1.8; 3.3)	0.72 (0.39; 1.33)	2.3 (1.6; 3.0)	1.35 (0.74; 2.43)	**2.6 (2.1; 3.1)**[c,b]	1.20 (0.66; 2.19)
Whole grains (serving/week)						
Satisfied	3.9 (2.7; 5.0)	[Ref]	3.5 (2.3; 4.6)	[Ref]	2.8 (1.9; 3.6)	[Ref]
Desire to Lose	3.8 (2.8; 4.8)	1.13 (0.71; 1.81)	2.9 (1.8; 4.0)	0.92 (0.57; 1.47)	2.7 (1.9; 3.6)	1.14 (0.69; 1.88)

Desire to Gain	3.7 (2.5; 5.0)	0.77 (0.45; 1.29)	4.0 (2.7; 5.2)	1.06 (0.65; 1.74)	3.1 (2.2; 4.0)	0.99 (0.60; 1.62)
Processed meat (serving/week)						
Satisfied	4.8 (4.0; 5.6)	[Ref]	3.9 (3.4; 4.4)	[Ref]	**3.5 (3.0; 3.9)**[b]	[Ref]
Desire to Lose	4.7 (4.1; 5.3)	0.91 (0.55; 1.51)	3.6 (3.1; 4.1)	0.77 (0.44; 1.34)	**3.2 (2.8; 3.6)**[c]	0.72 (0.37; 1.38)
Desire to Gain	5.7 (4.8; 6.6)	1.20 (0.71; 2.06)	4.0 (3.4; 4.6)	1.20 (0.68; 2.11)	**4.3 (3.8; 4.8)**[b,c]	1.33 (0.74; 2.38)
DQI-A						
Satisfied	57.2 (55.1; 59.4)	[Ref]	57.2 (54.8; 59.6)	[Ref]	59.5 (57.5; 61.5)	[Ref]
Desire to Lose	56.9 (55.1; 58.6)	1.18 (0.74; 1.90)	59.0 (56.7; 61.3)	1.36 (0.85; 2.20)	59.6 (57.6; 61.6)	0.81 (0.49; 1.35)
Desire to Gain	55.3 (53.0; 57.6)	0.83 (0.49; 1.39)	56.2 (53.5; 58.8)	1.02 (0.61; 1.70)	58.9 (56.8; 61.1)	0.73 (0.44; 1.20)

GIRLS

	**BASELINE** (n=626)		**2-YEAR FOLLOW-UP** (n=568)		**4-YEAR FOLLOW-UP** (n=508)	
	**mean (95% CI)** †	**OR (95% CI)** ‡	**mean (95% CI)** †	**OR (95% CI)** ‡	**mean (95% CI)** †	**OR (95% CI)** ‡
Fruits and vegetables (serving/day)						
Satisfied	2.4 (2.0; 2.8)	[Ref]	2.4 (2.2; 2.7)	[Ref]	2.3 (2.0; 2.6)	[Ref]
Desire to Lose	2.2 (1.8; 2.6)	0.83 (0.54; 1.30)	2.2 (1.9; 2.4)	**0.60 (0.38; 0.95)**	2.3 (2.0; 2.5)	1.17 (0.73; 1.86)
Desire to Gain	2.3 (1.8; 2.8)	0.85 (0.46; 1.55)	1.9 (1.4; 2.3)	0.53 (0.27; 1.05)	2.1 (1.7; 2.5)	0.66 (0.33; 1.31)
Fast food (serving/week)						
Satisfied	4.1 (3.6; 4.7)	[Ref]	3.8 (3.2; 4.4)	[Ref]	3.4 (2.9; 3.9)	[Ref]
Desire to Lose	3.8 (3.2; 4.4)	0.83 (0.53; 1.29)	3.6 (3.0; 4.3)	0.79 (0.51; 1.21)	3.0 (2.6; 3.4)	0.70 (0.43; 1.13)
Desire to Gain	4.0 (3.0; 5.0)	1.05 (0.58; 1.89)	4.5 (3.5; 5.6)	1.53 (0.84; 2.78)	3.9 (3.1; 4.6)	1.29 (0.69; 2.44)
Sugared and sweetened beverages (serving/week)						
Satisfied	12.8 (10.7; 14.8)	[Ref]	10.9 (9.6; 12.2)	[Ref]	9.5 (7.6; 11.4)	[Ref]
Desire to Lose	12.2 (10.1; 14.4)	0.81 (0.51; 1.28)	9.9 (8.6; 11.3)	0.74 (0.48; 1.15)	7.6 (5.9; 9.3)	0.71 (0.44; 1.13)
Desire to Gain	14.6 (11.6; 17.6)	1.31 (0.73; 2.36)	10.9 (8.3; 13.4)	1.06 (0.58; 1.95)	10.3 (7.8; 12.7)	1.15 (0.61; 2.17)
Sweets (serving/week)						
Satisfied	10.7 (8.9; 12.5)	[Ref]	11.7 (9.1; 14.3)	[Ref]	7.6 (6.4; 8.8)	[Ref]
Desire to Lose	11.1 (9.3; 13.0)	0.86 (0.56; 1.32)	9.3 (6.6; 12.0)	0.73 (0.47; 1.12)	6.9 (5.9; 7.9)	0.70 (0.45; 1.10)
Desire to Gain	11.6 (8.8; 14.5)	**1.73 (1.00; 2.97)**	13.2 (9.0; 17.0)	1.35 (0.75; 2.43)	9.5 (7.6; 11.3)	1.09 (0.59; 1.99)
Snacks (serving/week)						
Satisfied	2.1 (1.5; 2.8)	[Ref]	2.4 (1.6; 3.1)	[Ref]	**1.3 (1.0; 1.6)**[b]	[Ref]
Desire to Lose	2.1 (1.5; 2.8)	1.05 (0.59; 1.90)	2.0 (1.3; 2.8)	**0.54 (0.30; 0.99)**	1.4 (1.2; 1.7)	1.44 (0.61; 3.39)
Desire to Gain	2.8 (2.0; 3.7)	1.76 (0.86; 3.60)	2.6 (1.6; 3.6)	0.88 (0.40; 1.93)	**1.9 (1.4; 2.3)**[b]	2.31 (0.86; 6.21)

Nuts (serving/week)						
Satisfied	2.2 (1.5; 2.8)	[Ref]	2.5 (1.7; 3.3)	[Ref]	1.8 (1.2; 2.4)	[Ref]
Desire to Lose	2.0 (1.3; 2.6)	0.75 (0.42; 1.33)	2.5 (1.7; 3.4)	0.90 (0.53; 1.51)	1.8 (1.2; 2.4)	0.88 (0.49; 1.58)
Desire to Gain	2.1 (1.2; 2.9)	0.80 (0.37; 1.73)	1.9 (0.7; 3.0)	0.48 (0.21; 1.10)	2.2 (1.4; 2.9)	1.69 (0.81; 3.51)
Whole grains (serving/week)						
Satisfied	3.5 (2.3; 4.7)	[Ref]	3.3 (2.6; 4.0)	[Ref]	2.9 (2.2; 3.6)	[Ref]
Desire to Lose	3.9 (2.7; 5.1)	1.24 (0.80; 1.92)	3.7 (3.0; 4.4)	1.18 (0.78; 1.79)	3.4 (2.9; 4.0)	0.96 (0.60; 1.53)
Desire to Gain	3.5 (1.9; 5.1)	0.97 (0.53; 1.80)	2.5 (1.0; 3.9)	0.76 (0.39; 1.47)	2.5 (1.4; 3.6)	0.68 (0.34; 1.36)
Processed meat (serving/week)						
Satisfied	4.4 (3.8; 5.0)	[Ref]	3.7 (3.3; 4.2)	[Ref]	3.5 (2.9; 4.0)	[Ref]
Desire to Lose	4.5 (3.9; 5.1)	1.01 (0.62; 1.63)	4.1 (3.6; 4.5)	1.04 (0.63; 1.71)	3.7 (3.2; 4.1)	1.31 (0.76; 2.24)
Desire to Gain	5.2 (4.1; 6.3)	1.04 (0.54; 1.99)	4.2 (3.3; 5.1)	1.26 (0.61; 2.63)	4.6 (3.7; 5.4)	1.68 (0.80; 3.51)
DQI-A						
Satisfied	59.2 (57.5; 60.9)	[Ref]	59.6 (57.5; 61.8)	[Ref]	60.4 (58.2; 62.5)	[Ref]
Desire to Lose	58.2 (56.4; 59.9)	0.96 (0.62; 1.49)	60.7 (58.5; 62.9)	0.88 (0.56; 1.38)	**61.3 (59.5; 63.2)**[c]	1.21 (0.77; 1.92)
Desire to Gain	58.6 (55.8; 61.4)	1.08 (0.60; 1.96)	57.2 (53.7; 60.8)	0.74 (0.38; 1.44)	**56.8 (53.6; 60.0)**[c]	0.70 (0.36; 1.37)

BIS, body image satisfaction; DQI-A, diet quality index for adolescents (ranged from -33/100%). † Estimated marginal means (95% confidence intervals (CI) obtained from multilevel linear mixed-effects models. Fixed effects were body image satisfaction, age, nutritional status (underweight/normal weight/overweight/obesity), educational level (low/intermediate/high/unknown), migrant background (yes/no/unknown), moderate-to-vigorous physical activity, sexual maturity status, and randomization group (long term intervention/short term intervention/control). Region (Madrid/Barcelona) and schools were handled as random effects. Significant differences after Bonferroni correction (p≤0.05) are presented in bold: a, significant differences between desire to lose weight vs satisfied; b, significant differences between desire to gain weight vs satisfied; c, significant differences between desire to gain weight vs desire to lose weight. ‡ Odds ratios (OR) (95% CI) of being in the highest consumption tertile were calculated by logistic regression using the same adjusted model. High food consumption was defined as the highest gender-specific tertile of food consumption.

Supplemental Table S5. Mean unadjusted food intake and OR for the highest food consumption tertile by BIS and gender.

BOYS								
	**BASELINE** (n=674)		**2-YEAR FOLLOW-UP** (n=627)		**4-YEAR FOLLOW-UP** (n=576)		**REPEATED MEASURES***	
	**mean (95% CI) †**	**OR (95% CI) ‡**	**mean (95% CI) †**	**OR (95% CI) ‡**	**mean (95% CI) †**	**OR (95% CI) ‡**	**mean (95% CI) †**	**OR (95% CI) ‡**
Fruits and vegetables (serving/day)								
Satisfied	2.5 (2.2; 2.8)	[Ref]	2.0 (1.8; 2.3)	[Ref]	2.1 (1.8; 2.3)	[Ref]	2.1 (1.9; 2.3)	[Ref]
Desire to Lose	2.4 (2.1; 2.6)	0.78 (0.53; 1.16)	2.1 (1.8; 2.3)	1.05 (0.70; 1.55)	2.0 (1.7; 2.3)	0.93 (0.60; 1.43)	2.2 (2.0; 2.4)	1.00 (0.71; 1.42)
Desire to Gain	2.0 (1.7; 2.4)	**0.61 (0.38; 0.98)**	1.9 (1.6; 2.2)	0.68 (0.42; 1.09)	2.0 (1.7; 2.3)	0.90 (0.57; 1.42)	2.0 (1.8; 2.3)	0.80 (0.54; 1.19)
Fast food (serving/week)								
Satisfied	5.2 (3.9; 6.4)	[Ref]	4.3 (3.6; 4.9)	[Ref]	**3.7 (3.1; 4.4)**[b]	[Ref]	**4.4 (3.8; 5.0)**[b]	[Ref]
Desire to Lose	5.3 (4.4; 6.2)	0.94 (0.57; 1.55)	**3.7 (3.1; 4.3)**[c]	0.79 (0.45; 1.38)	**3.3 (2.7; 3.9)**[c]	0.81 (0.52; 1.27)	**4.4 (3.9; 5.0)**[c]	0.80 (0.57; 1.11)
Desire to Gain	5.8 (4.5; 7.0)	1.50 (0.86; 2.61)	**5.1 (4.3; 5.9)**[c]	1.37 (0.76; 2.46)	**5.2 (4.6; 5.9)**[b,c]	**1.90 (1.22; 2.96)**	**5.4 (4.8; 6.1)**[b,c]	**1.81 (1.28; 2.55)**
Sugared and sweetened beverages (serving/week)								
Satisfied	17.6 (14.4; 20.9)	[Ref]	**14.2 (12.2; 16.1)**[a]	[Ref]	10.2 (8.5; 11.9)	[Ref]	14.1 (12.2; 16.0)	[Ref]
Desire to Lose	16.5 (13.8; 19.1)	0.78 (0.52; 1.19)	**11.4 (9.6; 13.2)**[a]	**0.58 (0.38; 0.88)**	**8.8 (7.2; 10.4)**[c]	**0.60 (0.38; 0.93)**	13.4 (11.6; 15.2)	**0.55 (0.39; 0.78)**
Desire to Gain	17.6 (14.3; 20.9)	1.26 (0.78; 2.03)	13.6 (11.4; 15.8)	1.05 (0.67; 1.65)	**12.3 (10.6; 14.0)**[c]	1.13 (0.73; 1.75)	14.4 (12.4; 16.4)	1.06 (0.74; 1.53)
Sweets (serving/week)								
Satisfied	12.4 (10.0; 14.8)	[Ref]	**9.5 (6.5; 12.4)**[b]	[Ref]	**7.6 (6.0; 9.1)**[b]	[Ref]	**9.8 (8.5; 11.2)**[b]	[Ref]
Desire to Lose	10.5 (8.8; 12.3)	0.68 (0.45; 1.03)	**9.1 (6.2; 12.0)**[c]	0.74 (0.48; 1.15)	**5.4 (3.9; 6.9)**[c]	**0.43 (0.27; 0.69)**	**9.2 (8.0; 10.4)**[c]	**0.59 (0.42; 0.82)**
Desire to Gain	13.4 (11.0; 15.8)	1.32 (0.83; 2.10)	**13.4 (10.3; 16.5)**[b,c]	**2.50 (1.58; 3.97)**	**10.3 (8.7; 11.8)**[b,c]	**1.64 (1.06; 2.52)**	**11.7 (10.3; 13.2)**[b,c]	**1.94 (1.37; 2.76)**
Snacks (serving/week)								
Satisfied	2.4 (1.8; 2.9)	[Ref]	2.1 (1.4; 2.7)	[Ref]	1.5 (1.1; 1.9)	[Ref]	**2.0 (1.5; 2.5)**[b]	[Ref]
Desire to Lose	2.1 (1.7; 2.6)	0.85 (0.51; 1.44)	**1.7 (1.1; 2.3)**[c]	0.86 (0.48; 1.52)	**1.4 (1.0; 1.7)**[c]	0.71 (0.33; 1.53)	**1.9 (1.5; 2.4)**[c]	0.99 (0.63; 1.56)
Desire to Gain	2.8 (2.2; 3.4)	**1.75 (1.01; 3.05)**	**2.7 (2.0; 3.3)**[c]	**1.98 (1.11; 3.52)**	**2.1 (1.6; 2.5)**[c]	1.58 (0.79; 3.16)	**2.5 (2.0; 3.0)**[b,c]	**2.07 (1.30; 3.30)**
Nuts (serving/week)								
Satisfied	2.6 (1.9; 3.2)	[Ref]	2.1 (1.5; 2.8)	[Ref]	1.7 (1.3; 2.2)	[Ref]	2.1 (1.7; 2.5)	[Ref]
Desire to Lose	2.2 (1.8; 2.7)	0.79 (0.49; 1.27)	1.9 (1.3; 2.5)	0.97 (0.58; 1.62)	**1.5 (1.1; 1.9)**[c]	0.68 (0.38; 1.23)	**2.0 (1.6; 2.3)**[c]	0.91 (0.62; 1.32)
Desire to Gain	2.4 (1.8; 3.0)	0.69 (0.39; 1.22)	2.7 (2.0; 3.4)	1.36 (0.78; 2.37)	**2.4 (2.0; 2.9)**[c]	1.26 (0.73; 2.19)	**2.5 (2.1; 2.9)**[c]	1.18 (0.78; 1.79)

Whole grains (serving/week)								
Satisfied	3.8 (2.5; 5.0)	[Ref]	3.5 (2.1; 4.9)	[Ref]	2.9 (2.0; 3.7)	[Ref]	3.4 (2.3; 4.4)	[Ref]
Desire to Lose	4.0 (3.0; 5.1)	1.13 (0.75; 1.70)	3.2 (1.9; 4.6)	0.96 (0.65; 1.43)	2.7 (1.9; 3.5)	0.87 (0.56; 1.35)	3.5 (2.4; 4.5)	1.01 (0.73; 1.41)
Desire to Gain	3.6 (2.4; 4.9)	0.84 (0.51; 1.38)	3.7 (2.2; 5.1)	0.93 (0.59; 1.47)	2.9 (2.0; 3.8)	0.86 (0.55; 1.36)	3.6 (2.5; 4.7)	0.95 (0.66; 1.37)
Processed meat (serving/week)								
Satisfied	4.7 (3.9; 5.5)	[Ref]	3.9 (3.4; 4.4)	[Ref]	**3.5 (3.1; 3.9)**[b]	[Ref]	4.0 (3.6; 4.3)	[Ref]
Desire to Lose	4.8 (4.3; 5.4)	1.10 (0.71; 1.71)	3.5 (3.1; 4.0)	0.91 (0.57; 1.46)	**3.2 (2.8; 3.6)**[c]	0.64 (0.35; 1.14)	4.0 (3.7; 4.3)	1.02 (0.74; 1.41)
Desire to Gain	5.5 (4.7; 6.3)	1.16 (0.70; 1.93)	3.9 (3.3; 4.4)	1.08 (0.63; 1.84)	**4.3 (3.9; 4.7)**[b,c]	1.30 (0.76; 2.25)	4.5 (4.2; 4.9)	1.23 (0.86; 1.76)
DQI-A								
Satisfied	57.6 (55.2; 60.1)	[Ref]	57.4 (55.1; 59.6)	[Ref]	60.3 (58.0; 62.6)	[Ref]	57.3 (55.5; 59.2)	[Ref]
Desire to Lose	56.6 (54.6; 58.7)	0.95 (0.64; 1.42)	58.5 (56.3; 60.6)	1.11 (0.74; 1.67)	58.9 (56.7; 61.1)	0.66 (0.43; 1.01)	57.7 (55.9; 59.5)	1.09 (0.75; 1.59)
Desire to Gain	54.9 (52.4; 57.3)	0.70 (0.43; 1.14)	55.7 (53.2; 58.2)	0.97 (0.61; 1.56)	58.4 (56.1; 60.7)	**0.58 (0.37; 0.92)**	56.9 (55.0; 58.9)	0.77 (0.51; 1.18)

GIRLS

	**BASELINE** (n=632)		**2-YEAR FOLLOW-UP** (n=576)		**4-YEAR FOLLOW-UP** (n=529)		**REPEATED MEASURES***	
	**mean (95% CI)** †	**OR (95% CI)** ‡	**mean (95% CI)** †	**OR (95% CI)** ‡	**mean (95% CI)** †	**OR (95% CI)** ‡	**mean (95% CI)** †	**OR (95% CI)** ‡
Fruits and vegetables (serving/day)								
Satisfied	2.4 (2.1; 2.8)	[Ref]	2.4 (2.1; 2.6)	[Ref]	2.3 (2.0; 2.6)	[Ref]	2.3 (2.1; 2.6)	[Ref]
Desire to Lose	2.3 (1.9; 2.6)	0.81 (0.56; 1.17)	2.2 (2.0; 2.5)	0.74 (0.50; 1.09)	2.3 (2.0; 2.6)	1.12 (0.74; 1.70)	2.3 (2.1; 2.5)	0.91 (0.65; 1.26)
Desire to Gain	2.1 (1.6; 2.6)	0.69 (0.39; 1.21)	1.9 (1.4; 2.3)	0.56 (0.30; 1.03)	2.1 (1.6; 2.5)	0.70 (0.36; 1.34)	2.1 (1.8; 2.4)	0.69 (0.43; 1.13)
Fast food (serving/week)								
Satisfied	4.1 (3.4; 4.7)	[Ref]	3.9 (3.2; 4.5)	[Ref]	3.5 (2.9; 4.0)	[Ref]	3.9 (3.5; 4.4)	[Ref]
Desire to Lose	3.9 (3.2; 4.5)	0.89 (0.61; 1.32)	3.6 (2.9; 4.2)	0.75 (0.51; 1.12)	**2.9 (2.5; 3.3)**[c]	**0.58 (0.37; 0.91)**	3.6 (3.1; 4.0)	0.74 (0.54; 1.02)
Desire to Gain	4.4 (3.3; 5.4)	1.19 (0.69; 2.05)	4.7 (3.7; 5.7)	1.58 (0.91; 2.76)	**4.1 (3.3; 4.9)**[c]	1.39 (0.77; 2.52)	4.3 (3.6; 4.9)	1.44 (0.92; 2.26)
Sugared and sweetened beverages (serving/week)								
Satisfied	12.3 (10.2; 14.5)	[Ref]	11.1 (9.5; 12.7)	[Ref]	9.6 (7.9; 11.3)	[Ref]	11.4 (9.8; 13.0)	[Ref]
Desire to Lose	12.8 (10.6; 14.9)	0.84 (0.57; 1.25)	9.7 (8.1; 11.3)	0.73 (0.50; 1.08)	7.6 (6.1; 9.1)	0.68 (0.44; 1.04)	10.5 (9.0; 12.1)	0.76 (0.55; 1.05)
Desire to Gain	14.1 (11.1; 17.1)	1.41 (0.82; 2.42)	11.6 (9.0; 14.2)	1.13 (0.65; 1.96)	9.8 (7.3; 12.2)	1.02 (0.56; 1.86)	11.5 (9.5; 13.5)	1.12 (0.71; 1.76)
Sweets (serving/week)								

Satisfied	10.8 (8.9; 12.7)	[Ref]	**11.7 (9.4; 14.1)**[a]	[Ref]	7.9 (6.7; 9.1)	[Ref]	10.6 (9.1; 12.0)	[Ref]
Desire to Lose	10.9 (9.0; 12.8)	0.86 (0.59; 1.25)	**8.6 (6.3; 11.0)**[a,c]	**0.62 (0.42; 0.91)**	**6.7 (5.7; 7.7)**[c]	**0.60 (0.40; 0.90)**	9.1 (7.7; 10.6)	**0.66 (0.49; 0.89)**
Desire to Gain	12.3 (9.5; 15.2)	**1.86 (1.11; 3.11)**	**13.8 (10.2; 17.4)**[c]	1.47 (0.86; 2.52)	**9.8 (7.9; 11.6)**[c]	1.01 (0.57; 1.79)	11.2 (9.2; 13.2)	1.31 (0.87; 1.99)
Snacks (serving/week)								
Satisfied	**2.0 (1.6; 2.4)**[b]	[Ref]	2.4 (1.7; 3.0)	[Ref]	1.4 (1.1; 1.7)	[Ref]	**2.0 (1.6; 2.4)**[b]	[Ref]
Desire to Lose	**1.9 (1.5; 2.3)**[c]	1.14 (0.68; 1.91)	1.9 (1.2; 2.6)	**0.58 (0.34; 0.98)**	1.4 (1.2; 1.7)	0.98 (0.46; 2.06)	**1.8 (1.4; 2.2)**[c]	0.71 (0.47; 1.08)
Desire to Gain	**3.1 (2.4; 3.8)**[b,c]	**2.32 (1.22; 4.42)**	2.5 (1.6; 3.4)	0.93 (0.46; 1.90)	1.9 (1.4; 2.3)	1.89 (0.76; 4.67)	**2.5 (2.0; 3.0)**[b,c]	1.52 (0.90; 2.59)
Nuts (serving/week)								
Satisfied	2.2 (1.5; 2.8)	[Ref]	2.6 (1.8; 3.3)	[Ref]	1.8 (1.2; 2.3)	[Ref]	2.2 (1.6; 2.8)	[Ref]
Desire to Lose	2.0 (1.3; 2.6)	0.68 (0.42; 1.11)	2.3 (1.5; 3.1)	0.77 (0.48; 1.24)	1.8 (1.3; 2.3)	0.98 (0.58; 1.66)	2.1 (1.5; 2.7)	0.80 (0.57; 1.12)
Desire to Gain	2.1 (1.2; 3.0)	0.75 (0.36; 1.53)	2.2 (1.1; 3.3)	0.53 (0.24; 1.15)	2.1 (1.4; 2.8)	1.60 (0.80; 3.21)	2.2 (1.5; 2.9)	0.87 (0.53; 1.42)
Whole grains (serving/week)								
Satisfied	3.5 (2.2; 4.7)	[Ref]	3.3 (2.5; 4.0)	[Ref]	3.0 (2.2; 3.7)	[Ref]	3.4 (2.6; 4.1)	[Ref]
Desire to Lose	4.0 (2.8; 5.3)	1.37 (0.93; 2.00)	3.8 (3.0; 4.5)	1.39 (0.96; 2.01)	3.6 (2.9; 4.2)	1.02 (0.67; 1.56)	3.8 (3.0; 4.5)	1.26 (0.93; 1.71)
Desire to Gain	3.3 (1.7; 4.8)	0.91 (0.51; 1.63)	2.2 (0.9; 3.6)	0.69 (0.37; 1.27)	2.4 (1.3; 3.6)	0.69 (0.35; 1.33)	2.9 (1.9; 3.8)	0.70 (0.44; 1.11)
Processed meat (serving/week)								
Satisfied	4.4 (3.8; 5.0)	[Ref]	3.8 (3.4; 4.2)	[Ref]	3.4 (2.7; 4.0)	[Ref]	3.9 (3.6; 4.3)	[Ref]
Desire to Lose	4.5 (3.9; 5.1)	1.09 (0.72; 1.64)	4.0 (3.6; 4.5)	1.14 (0.73; 1.80)	3.6 (3.1; 4.2)	1.23 (0.75; 2.02)	4.1 (3.8; 4.5)	1.24 (0.88; 1.73)
Desire to Gain	5.5 (4.4; 6.5)	1.14 (0.63; 2.06)	4.0 (3.2; 4.9)	1.00 (0.50; 1.99)	4.3 (3.4; 5.2)	1.43 (0.71; 2.88)	4.6 (4.0; 5.1)	1.14 (0.70; 1.84)
DQI-A								
Satisfied	59.4 (57.2; 61.5)	[Ref]	59.3 (56.9; 61.8)	[Ref]	60.3 (57.9; 62.8)	[Ref]	59.3 (57.5; 61.0)	[Ref]
Desire to Lose	58.3 (56.2; 60.4)	0.91 (0.63; 1.31)	**61.1 (58.6; 63.6)**[c]	1.04 (0.70; 1.53)	61.1 (58.9; 63.2)	1.11 (0.73; 1.67)	59.7 (58.0; 61.5)	1.10 (0.77; 1.57)
Desire to Gain	56.9 (53.8; 59.9)	0.85 (0.49; 1.47)	**56.6 (53.0; 60.2)**[c]	0.64 (0.35; 1.19)	57.2 (53.7; 60.6)	0.81 (0.43; 1.51)	57.9 (55.7; 60.2)	0.82 (0.48; 1.38)

BIS, body image satisfaction; DQI-A, diet quality index for adolescents (ranged from -33/100%). † Unadjusted estimated marginal means (95% confidence intervals (CI)) obtained from multilevel linear mixed-effects models. Region (Madrid/Barcelona) and schools were handled as random effects. Significant differences after Bonferroni correction (p≤0.05) are presented in bold: a, significant differences between desire to lose weight vs satisfied; b, significant differences between desire to gain weight vs satisfied; c, significant differences between desire to gain weight vs desire to lose. ‡ Odds ratios (OR) (95% CI) of being in the highest consumption tertile or in the highest emotional eating or self-esteem categories were calculated by logistic regression using the same adjusted model. * Estimated marginal means (95% CI) and OR were obtained from mixed-effect model for repeated measures at baseline, 2- and 4-year follow-up using the same adjusted model with the addition of participants as random effects. High food consumption was defined as the highest gender-specific tertile of food consumption.

Supplemental Table S6. Emotional management during adolescence at baseline, 2- and 4-year follow-up by BIS and gender.

	BOYS					
	BASELINE (n=674)		2-YEAR FOLLOW-UP (n=620)		4-YEAR FOLLOW-UP (n=563)	
	**mean (95% CI) †**	**OR (95% CI) ‡**	**mean (95% CI) †**	**OR (95% CI) ‡**	**mean (95% CI) †**	**OR (95% CI) ‡**
Self-esteem						
Satisfied	**15.9 (15.6; 16.2)[a]**	[Ref]	**15.5 (15.2; 15.8)[a,b]**	[Ref]	**15.3 (15.0; 15.6)[a,b]**	[Ref]
Desire to Lose	**15.4 (15.2; 15.7)[a]**	0.78 (0.28; 2.14)	**14.8 (14.5; 15.1)[a]**	0.71 (0.45; 1.14)	**14.3 (14.0; 14.6)[a]**	**0.42 (0.25; 0.72)**
Desire to Gain	15.7 (15.3; 16.0)	**2.66 (1.05; 6.75)**	**14.9 (14.5; 15.3)[b]**	0.71 (0.44; 1.17)	**14.2 (13.9; 14.6)[b]**	**0.36 (0.21; 0.62)**
Emotional Eating						
Satisfied	4.5 (4.0; 4.9)	[Ref]	4.3 (4.0; 4.6)	[Ref]	4.5 (4.2; 4.9)	[Ref]
Desire to Lose	4.5 (4.1; 4.9)	0.85 (0.52; 1.41)	4.7 (4.5; 5.0)	**1.89 (1.18; 3.02)**	4.8 (4.5; 5.2)	1.12 (0.61; 2.08)
Desire to Gain	4.8 (4.3; 5.2)	1.44 (0.86; 2.40)	4.6 (4.2; 4.9)	1.25 (0.75; 2.08)	4.9 (4.6; 5.3)	1.68 (0.92; 3.05)
	**GIRLS**					
	BASELINE (n=625)		2-YEAR FOLLOW-UP (n=569)		4-YEAR FOLLOW-UP (n=508)	
	**mean (95% CI) †**	**OR (95% CI) ‡**	**mean (95% CI) †**	**OR (95% CI) ‡**	**mean (95% CI) †**	**OR (95% CI) ‡**
Self-esteem						
Satisfied	15.7 (15.4; 15.9)	[Ref]	**15.1 (14.7; 15.4)[a]**	[Ref]	**14.4 (13.8; 14.9)[a,b]**	[Ref]
Desire to Lose	15.3 (15.0; 15.5)	1.39 (0.56; 3.43)	**13.8 (13.4; 14.2)[a]**	**0.41 (0.25; 0.68)**	**12.9 (12.5; 13.4)[a]**	**0.41 (0.26; 0.64)**
Desire to Gain	15.6 (15.2; 16.0)	1.55 (0.48; 5.00)	14.4 (13.8; 15.0)	**0.37 (0.17; 0.79)**	**13.2 (12.5; 13.9)[b]**	**0.51 (0.27; 0.97)**
Emotional Eating						
Satisfied	4.5 (4.2; 4.8)	[Ref]	**5.1 (4.8; 5.4)[b]**	[Ref]	**5.2 (4.9; 5.6)[a,b]**	[Ref]
Desire to Lose	4.6 (4.3; 4.9)	0.94 (0.61; 1.46)	5.5 (5.2; 5.8)	0.95 (0.61; 1.48)	**6.3 (6.0; 6.6)[a]**	**2.24 (1.32; 3.83)**
Desire to Gain	4.9 (4.4; 5.4)	1.29 (0.72; 2.30)	**6.0 (5.4; 6.6)[b]**	1.73 (0.95; 3.15)	**6.5 (5.9; 7.1)[b]**	**3.10 (1.54; 6.23)**

BIS, body image satisfaction. Emotional eating scale ranged 1-12 points, and self-esteem score ranged 1-20 points. The number of participants varied due to data availability. † Estimated marginal means (95% confidence intervals (CI) obtained from multilevel linear mixed-effects models. Fixed effects were body image satisfaction, age, nutritional status (underweight/normal weight/overweight/obesity), educational level (low/intermediate/high/unknown), migrant background (yes/no/unknown), randomization group (long term intervention/short term intervention/control), moderate-to-vigorous physical activity and sexual maturity status. Region (Madrid/Barcelona) and schools were handled as random effects. Significant differences after Bonferroni correction ($p \leq 0.05$) are presented in bold: a, significant differences between desire to lose weight vs satisfied; b, significant differences between desire to gain weight vs satisfied; c, significant differences between desire to gain weight vs desire to lose weight. ‡ Odds ratios (OR) (95% CI) of being in the highest emotional eating or self-esteem categories were calculated by logistic regression using the same adjusted model.

Supplemental Table S7. Mean unadjusted emotional management scores and OR for the highest emotional management tertile by BIS and gender.

BOYS

	**BASELINE** (n=676)		**2-YEAR FOLLOW-UP** (n=629)		**4-YEAR FOLLOW-UP** (n=578)		**REPEATED MEASURES***	
	**mean (95% CI) †**	**OR (95% CI) ‡**	**mean (95% CI) †**	**OR (95% CI) ‡**	**mean (95% CI) †**	**OR (95% CI) ‡**	**mean (95% CI) †**	**OR (95% CI) ‡**
Self-esteem								
Satisfied	**16.0 (15.7; 16.3)[a]**	[Ref]	**15.6 (15.3; 15.9)[a,b]**	[Ref]	**15.3 (15.0; 15.6)[a,b]**	[Ref]	**15.5 (15.3; 15.7)[a,b]**	[Ref]
Desire to Lose	**15.4 (15.2; 15.6)[a]**	1.13 (0.48; 2.65)	**14.7 (14.4; 14.9)[a]**	**0.59 (0.40; 0.89)**	**14.2 (13.9; 14.5)[a]**	**0.41 (0.26; 0.66)**	**14.9 (14.8; 15.1)[a]**	**0.48 (0.36; 0.65)**
Desire to Gain	15.7 (15.5; 16.0)	2.33 (0.97; 5.61)	**15.0 (14.7; 15.4)[b]**	0.76 (0.48; 1.20)	**14.3 (14.0; 14.7)[b]**	**0.37 (0.22; 0.61)**	**15.0 (14.8; 15.2)[b]**	**0.66 (0.47; 0.91)**
Emotional Eating								
Satisfied	4.3 (3.8; 4.8)	[Ref]	**4.2 (3.9; 4.5)[a]**	[Ref]	**4.5 (4.2; 4.9)[a]**	[Ref]	**4.4 (4.1; 4.8)[a]**	[Ref]
Desire to Lose	4.6 (4.1; 5.0)	1.17 (0.77; 1.79)	**4.8 (4.6; 5.1)[a]**	**2.03 (1.35; 3.07)**	**5.1 (4.7; 5.4)[a]**	1.61 (0.95; 2.75)	**4.7 (4.4; 5.0)[a]**	**1.64 (1.15; 2.35)**
Desire to Gain	4.7 (4.1; 5.2)	1.59 (0.98; 2.57)	4.5 (4.2; 4.8)	1.40 (0.86; 2.27)	4.8 (4.5; 5.2)	1.68 (0.97; 2.90)	**4.7 (4.4; 5.0)**	**1.59 (1.08; 2.36)**

GIRLS

	**BASELINE** (n=631)		**2-YEAR FOLLOW-UP** (n=577)		**4-YEAR FOLLOW-UP** (n=530)		**REPEATED MEASURES***	
	**mean (95% CI) †**	**OR (95% CI) ‡**	**mean (95% CI) †**	**OR (95% CI) ‡**	**mean (95% CI) †**	**OR (95% CI) ‡**	**mean (95% CI) †**	**OR (95% CI) ‡**
Self-esteem								
Satisfied	**15.7 (15.4; 15.9)[a]**	[Ref]	**15.1 (14.7; 15.4)[a]**	[Ref]	**14.4 (13.9; 14.8)[a,b]**	[Ref]	**15.1 (14.9; 15.3)[a,b]**	[Ref]
Desire to Lose	**15.3 (15.0; 15.5)[a]**	1.62 (0.75; 3.49)	**13.7 (13.4; 14.1)[a,c]**	**0.39 (0.25; 0.62)**	**12.8 (12.4; 13.2)[a]**	**0.40 (0.27; 0.60)**	**14.0 (13.7; 14.2)[a,c]**	**0.57 (0.43; 0.76)**
Desire to Gain	15.7 (15.3; 16.1)	1.48 (0.50; 4.38)	**14.6 (14.0; 15.1)[c]**	**0.39 (0.19; 0.79)**	**13.3 (12.6; 13.9)[b]**	**0.52 (0.29; 0.94)**	**14.5 (14.2; 14.9)[b,c]**	**0.57 (0.37; 0.88)**
Emotional Eating								
Satisfied	4.5 (4.1; 4.8)	[Ref]	5.1 (4.8; 5.4)	[Ref]	**5.2 (4.8; 5.6)[a,b]**	[Ref]	**4.9 (4.6; 5.1)[a,b]**	[Ref]
Desire to Lose	4.7 (4.4; 5.0)	1.12 (0.77; 1.63)	5.5 (5.2; 5.8)	1.00 (0.67; 1.48)	**6.4 (6.1; 6.7)[a]**	**2.85 (1.74; 4.67)**	**5.5 (5.3; 5.7)[a]**	1.35 (0.99; 1.85)
Desire to Gain	4.8 (4.4; 5.3)	1.42 (0.83; 2.40)	5.9 (5.3; 6.4)	1.44 (0.82; 2.52)	**6.3 (5.7; 6.8)[b]**	**2.61 (1.34; 5.10)**	**5.6 (5.2; 5.9)[b]**	**1.84 (1.17; 2.89)**

BIS, body image satisfaction. Emotional eating scale ranged 1-12 points, and self-esteem score ranged 1-20 points. The number of participants varied due to data availability. † Unadjusted estimated marginal means (95% confidence intervals (CI)) obtained from multilevel linear mixed-effects models. Region (Madrid/Barcelona) and schools were handled as random effects. Significant differences after Bonferroni correction (p≤0.05) are presented in bold: a, significant differences between desire to lose weight vs satisfied; b, significant differences between desire to gain weight vs satisfied; c, significant differences between desire to gain weight vs desire to lose weight. ‡ Odds ratios (OR) (95% CI) of being in the highest emotional eating or self-esteem categories were calculated by logistic regression using the same adjusted model. * Estimated marginal means (95% CI) and OR were obtained from mixed-effects models for repeated measures, and OR (95% CI) for being in the highest emotional eating or self-esteem categories were obtained using the same adjusted model with the addition of participants as random effects. Emotional eaters and participants with high self-esteem were defined as those in the highest gender-specific tertile of emotional eating (ranged 1-12 points) and self-esteem score (ranged from 1-20 points), respectively.

Supplemental Table S8. Food consumption and emotional management by BIS trajectory and gender.

REPEATED MEASURES AT BASELINE, 2- AND 4-YEAR FOLLOW-UP				
	**BOYS**			
	**mean (95% CI)**			
	Always Satisfied	*Satisfied 2/3*	*Satisfied 1/3*	*Never Satisfied*
**DIETARY HABITS**				
**Fruits and vegetables (serving/day)**	2.4 (2.0; 2.8)	2.2 (1.9; 2.4)	2.0 (1.8; 2.3)	2.1 (1.9; 2.3)
**Fast food (serving/week)**	4.1 (3.0; 5.2)	4.2 (3.4; 5.0)	4.5 (3.8; 5.2)	4.4 (3.8; 4.9)
**Sugared and sweetened beverages (serving/week)**	13.8 (11.1; 16.5)	12.6 (10.7; 14.5)	13.5 (11.9; 15.2)	12.2 (10.8; 13.5)
**Sweets (serving/week)**	8.5 (6.2; 10.8)	8.5 (6.9; 10.1)	9.3 (7.9; 10.6)	9.1 (8.0; 10.1)
**Snacks (serving/week)**	2.0 (1.4; 2.7)	1.8 (1.3; 2.3)	2.0 (1.5; 2.5)	1.9 (1.5; 2.4)
**Nuts (serving/week)**	2.5 (1.8; 3.2)	1.9 (1.4; 2.4)	2.0 (1.6; 2.5)	2.1 (1.7; 2.4)
**Whole grains (serving/week)**	3.6 (2.2; 5.0)	3.1 (1.9; 4.2)	3.4 (2.3; 4.4)	3.2 (2.2; 4.2)
**Processed meat (serving/week)**	4.2 (3.5; 4.9)	4.1 (3.7; 4.6)	4.2 (3.8; 4.6)	4.0 (3.7; 4.3)
**DQI-A**	59.2 (56.3; 62.2)	59.3 (57.2; 61.5)	57.5 (55.6; 59.4)	57.9 (56.3; 59.5)
**EMOTIONAL MANAGEMENT**				
**Self-esteem**	**16.0 (15.6; 16.4)**[a,b,c]	**15.4 (15.1; 15.7)**[a,d]	**15.2 (15.0; 15.5)**[b,e]	**14.8 (14.6; 15.0)**[c,d,e]
**Emotional Eating**	4.4 (3.9; 4.9)	4.3 (3.9; 4.7)	4.4 (4.0; 4.8)	4.7 (4.4; 5.1)

	**GIRLS**			
	**mean (95% CI)**			
	Always Satisfied	*Satisfied 2/3*	*Satisfied 1/3*	*Never Satisfied*
**DIETARY HABITS**				
**Fruits and vegetables (serving/day)**	2.2 (1.9; 2.5)	2.4 (2.2; 2.7)	2.2 (2.0; 2.5)	2.1 (1.9; 2.3)
**Fast food (serving/week)**	3.9 (3.2; 4.6)	3.3 (2.7; 3.8)	3.4 (2.9; 4.0)	3.8 (3.3; 4.3)
**Sugared and sweetened beverages (serving/week)**	10.9 (8.9; 12.9)	10.4 (8.6; 12.1)	9.8 (8.1; 11.6)	10.5 (8.8; 12.1)
**Sweets (serving/week)**	10.4 (8.2; 12.6)	8.9 (7.0; 10.8)	9.2 (7.3; 11.1)	10.1 (8.3; 11.9)
**Snacks (serving/week)**	2.2 (1.6; 2.8)	1.7 (1.2; 2.2)	1.9 (1.4; 2.4)	2.0 (1.5; 2.5)
**Nuts (serving/week)**	2.5 (1.7; 3.2)	2.0 (1.3; 2.6)	2.2 (1.5; 2.9)	1.9 (1.3; 2.6)
**Whole grains (serving/week)**	3.2 (2.4; 4.0)	3.0 (2.4; 3.6)	3.1 (2.4; 3.7)	3.4 (2.8; 3.9)
**Processed meat (serving/week)**	3.9 (3.3; 4.5)	4.0 (3.5; 4.4)	4.1 (3.6; 4.6)	4.2 (3.8; 4.6)
**DQI-A**	59.3 (56.8; 61.8)	60.8 (58.7; 62.8)	60.6 (58.5; 62.7)	59.1 (57.3; 61.0)

EMOTIONAL MANAGEMENT				
**Self-esteem**	**15.2 (14.8; 15.6)[b,c]**	**14.8 (14.5; 15.1)[d]**	**14.3 (14.0; 14.6)[b]**	**13.9 (13.7; 14.2)[c,d]**
**Emotional Eating**	4.8 (4.4; 5.2)[c]	5.2 (4.9; 5.5)	**5.3 (5.0; 5.7)**	**5.6 (5.4; 5.9)[c]**

BIS, body image satisfaction; DQI-A, diet quality index for adolescents (ranged from -33/100%). Emotional eating scale ranged 1-12 points, and self-esteem score ranged 1-20 points. This analysis includes participants with data in the BIS variables in all three assessments. Estimated marginal means (95% confidence intervals (CI) were obtained from mixed-effect models for repeated measures at baseline, 2- and 4-year follow-up. Fixed effects were BIS trajectory, age, nutritional status (underweight/normal weight/overweight/obesity), educational level (low/intermediate/high/unknown), migrant background (yes/no/unknown), moderate-to-vigorous physical activity, sexual maturity status, and randomization group (long term intervention/short term intervention/control). Region (Madrid/Barcelona), schools, and participants were handled as random effects. Significant differences after Bonferroni correction ($p \leq 0.05$) are presented in bold: a, significant differences between always satisfied vs satisfied 2/3; b, always satisfied vs satisfied 1/3; c, always satisfied vs never satisfied; d, satisfied 2/3 vs never satisfied.

Supplemental Table S9. Odds of being in the highest food intake or emotional management category by BIS trajectory and gender.

REPEATED MEASURES AT BASELINE, 2- AND 4-YEAR FOLLOW-UP				
	**BOYS**			
	**OR (95% CI)**			
	Always Satisfied	*Satisfied 2/3*	*Satisfied 1/3*	*Never Satisfied*
**DIETARY HABITS**				
**Fruits and vegetables**	[Ref]	0.85 (0.40; 1.81)	0.78 (0.38; 1.62)	0.76 (0.38; 1.53)
**Fast food**	-	0.91 (0.45; 1.87)	1.14 (0.57; 2.25)	1.11 (0.58; 2.15)
**Sugared and sweetened beverages**	-	0.78 (0.38; 1.61)	0.99 (0.50; 1.97)	0.72 (0.37; 1.41)
**Sweets**	-	1.13 (0.56; 2.28)	1.35 (0.69; 2.65)	1.35 (0.70; 2.59)
**Snacks**	-	0.78 (0.30; 2.05)	0.97 (0.39; 2.42)	0.97 (0.40; 2.32)
**Nuts**	-	0.56 (0.29; 1.08)	0.70 (0.38; 1.30)	**0.55 (0.30; 1.00)**
**Whole grains**	-	0.82 (0.39; 1.71)	0.88 (0.44; 1.78)	0.72 (0.36; 1.42)
**Processed meat**	-	1.09 (0.56; 2.11)	1.00 (0.53; 1.91)	0.94 (0.50; 1.75)
**DQI-A**	-	0.96 (0.41; 2.24)	0.67 (0.29; 1.51)	0.67 (0.30; 1.47)
**EMOTIONAL MANAGEMENT**				
**Self-esteem**	[Ref]	0.72 (0.42; 1.25)	**0.47 (0.27; 0.81)**	**0.44 (0.26; 0.73)**
**Emotional Eating**	-	1.15 (0.51; 2.60)	1.21 (0.55; 2.65)	1.89 (0.89; 4.01)
	**GIRLS**			
	**OR (95% CI)**			
	Always Satisfied	*Satisfied 2/3*	*Satisfied 1/3*	*Never Satisfied*
**DIETARY HABITS**				
**Fruits and vegetables**	[Ref]	1.58 (0.84; 2.99)	1.14 (0.59; 2.19)	0.69 (0.36; 1.30)
**Fast food**	-	0.61 (0.34; 1.09)	**0.48 (0.26; 0.88)**	0.75 (0.42; 1.34)
**Sugared and sweetened beverages**	-	0.91 (0.50; 1.63)	0.64 (0.35; 1.17)	0.75 (0.42; 1.35)
**Sweets**	-	0.78 (0.45; 1.36)	0.77 (0.44; 1.36)	0.92 (0.53; 1.59)
**Snacks**	-	0.63 (0.32; 1.26)	0.78 (0.39; 1.53)	0.96 (0.50; 1.86)
**Nuts**	-	0.80 (0.44; 1.44)	0.91 (0.51; 1.65)	0.68 (0.37; 1.22)
**Whole grains**	-	0.72 (0.41; 1.25)	0.76 (0.43; 1.35)	1.00 (0.58; 1.73)
**Processed meat**	-	0.80 (0.44; 1.45)	1.15 (0.63; 2.08)	0.98 (0.55; 1.77)
**DQI-A**	-	1.19 (0.58; 2.41)	1.00 (0.48; 2.08)	0.70 (0.34; 1.44)

**EMOTIONAL MANAGEMENT**				
**Self-esteem**	[Ref]	0.78 (0.45; 1.35)	**0.49 (0.27; 0.88)**	**0.36 (0.20; 0.64)**
**Emotional Eating**	-	1.55 (0.82; 2.91)	1.41 (0.74; 2.68)	**1.93 (1.03; 3.60)**

BIS, body image satisfaction; DQI-A, diet quality index for adolescents. This analysis includes participants with data in the BIS variables in all three assessment. Odds ratios (OR) (95% CI) of being in the highest consumption tertile or for being in the highest emotional eating or self-esteem categories were calculated by logistic regression. Fixed effects were BIS trajectory, age, nutritional status (underweight/normal weight/overweight/obesity), educational level (low/intermediate/high/unknown), migrant background (yes/no/unknown), moderate-to-vigorous physical activity and sexual maturity status, and randomization group (long term intervention/short term intervention/control). Region (Madrid/Barcelona), schools, and participants were handled as random effects. Significant differences ($p \leq 0.05$) are presented in bold. High food consumption, emotional eaters and participants with high self-esteem were defined as those in the highest gender-specific tertile of food consumption, DQI-A (ranged from -33/100%), emotional eating (ranged 1-12 points) and self-esteem score (ranged 1-20 points), respectively.

Supplemental Table S10. Baseline characteristics of the participants included vs excluded from the analyses.

	**Included** (n=1125)	**Excluded** (n=190)	**p-value**
**Gender**, girls n (%)	544 (48.4%)	90 (47.4%)	0.801
**Age, years**, mean (SD)	12.5 (0.4)	12.7 (0.6)	**<0.001**
**Region,** n (%)			
Madrid	327 (29.1%)	93 (49.0%)	**<0.001**
Barcelona	798 (70.9%)	97 (51.1%)	
**Migrant background**, n (%)			
Spanish	761 (67.6%)	107 (56.3%)	**<0.001**
Migrant background	359 (31.9%)	69 (36.3%)	
Unknown	5 (0.4%)	14 (7.4%)	
**Parental education level**			
Low	214 (19.0%)	31 (16.3%)	**<0.001**
Intermediate	458 (40.7%)	75 (39.5%)	
High	450 (40.0%)	70 (36.8%)	
Unknown	3 (0.3%)	14 (7.4%)	
**Nutritional status**, n (%)			
Underweight	106 (9.5%)	23 (12.1%)	0.531
Normal weight	193 (17.3%)	37 (19.5%)	
Overweight	785 (70.3%)	124 (65.3%)	
Obesity	32 (2.9%)	6 (3.2%)	
**Body image satisfaction**, n (%)			
Satisfied	377 (33.7%)	60 (31.6%)	0.518
Desire to lose weight	540 (48.2%)	89 (46.8%)	
Desire to gain weight	203 (18.1%)	41 (21.6%)	
**DQI-A**, mean (SD)	58.1 (12.6)	55.1 (14.4)	**0.002**
Diet quality	43.4 (23.6)	35.3 (29.1)	**<0.001**
Dietary diversity	78.8 (16.9)	79.8 (15.5)	0.447
Dietary equilibrium	52.3 (11.8)	50.1 (12.2)	**0.017**
**Self-esteem**, mean (SD)	15.6 (1.8)	15.4 (2.0)	0.311
**Emotional eating**, mean (SD)	4.6 (2.0)	5.0 (2.3)	**0.008**

Values are expressed as mean (standard deviation) for continuous variables or as frequency (percentage) for categorical variables. Nutritional status was defined as age- and sex-adjusted body mass index percentiles according to Centers for Disease Control (CDC) standards[1]: underweight <5th percentile; normal weight ≥5th to <85th percentiles; overweight ≥85th to ≤95th percentiles; and obesity >95th percentile. DQI-A, diet quality index for adolescents (ranged from -33/100%). Dietary quality domain ranged from -100/100%, dietary diversity and dietary equilibrium domains ranged from 0/100%, emotional eating scale ranged 1-12 points, and self-esteem score ranged 1-20 points. P-values for gender differences were calculated by unpaired t-test or chi-square test, as appropriate. Significant differences (p≤0.05) are presented in bold.

[1] Kuczmarski RJ, Ogden CL, Guo SS, Grummer-Strawn LM, Flegal KM, Mei Z, et al. 2000 CDC Growth Charts for the United States: methods and development. Vital Health Stat 11. 2002(246):1-190.

Supplementary Figures

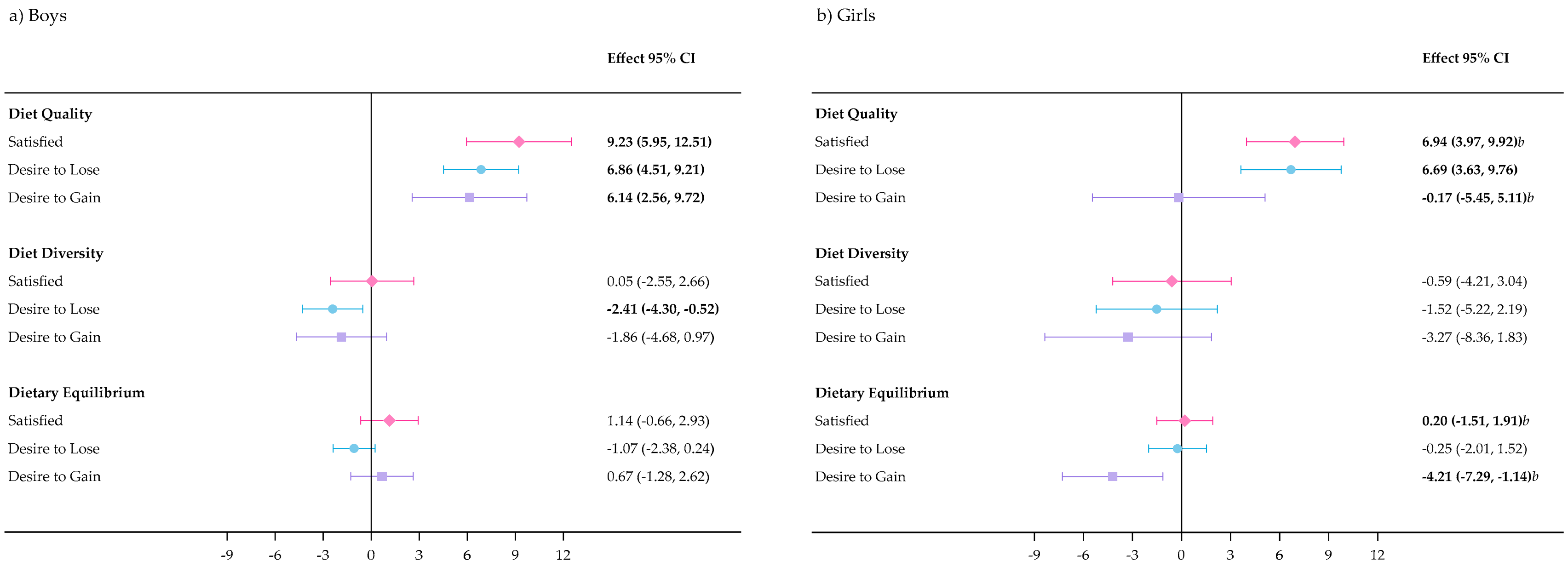

Supplemental Figure S1. Estimated mean change in DQI-A from baseline to 4-year follow-up by BIS and gender.

BIS, body image satisfaction; DQI-A, diet quality index for adolescents. Dietary quality domain scale ranged -100/100%, dietary diversity and dietary equilibrium domains scale ranged 0/100%, The number of participants varied due to data availability. Estimated marginal means (95% confidence intervals (CI)) obtained from multilevel linear mixed-effects models. Fixed effects were body image satisfaction, age, nutritional status (underweight/normal weight/overweight/obesity), educational level (low/intermediate/high/unknown), migrant background (yes/no/unknown), randomization group (long term intervention/short term intervention/control), moderate-to-vigorous physical activity, sexual maturity status, and DQI-A at baseline. Region (Madrid/Barcelona) and schools were handled as random effects. Significant differences (p≤0.05) are presented in bold. a, significant differences between satisfied vs desire to gain weight.

a) Boys

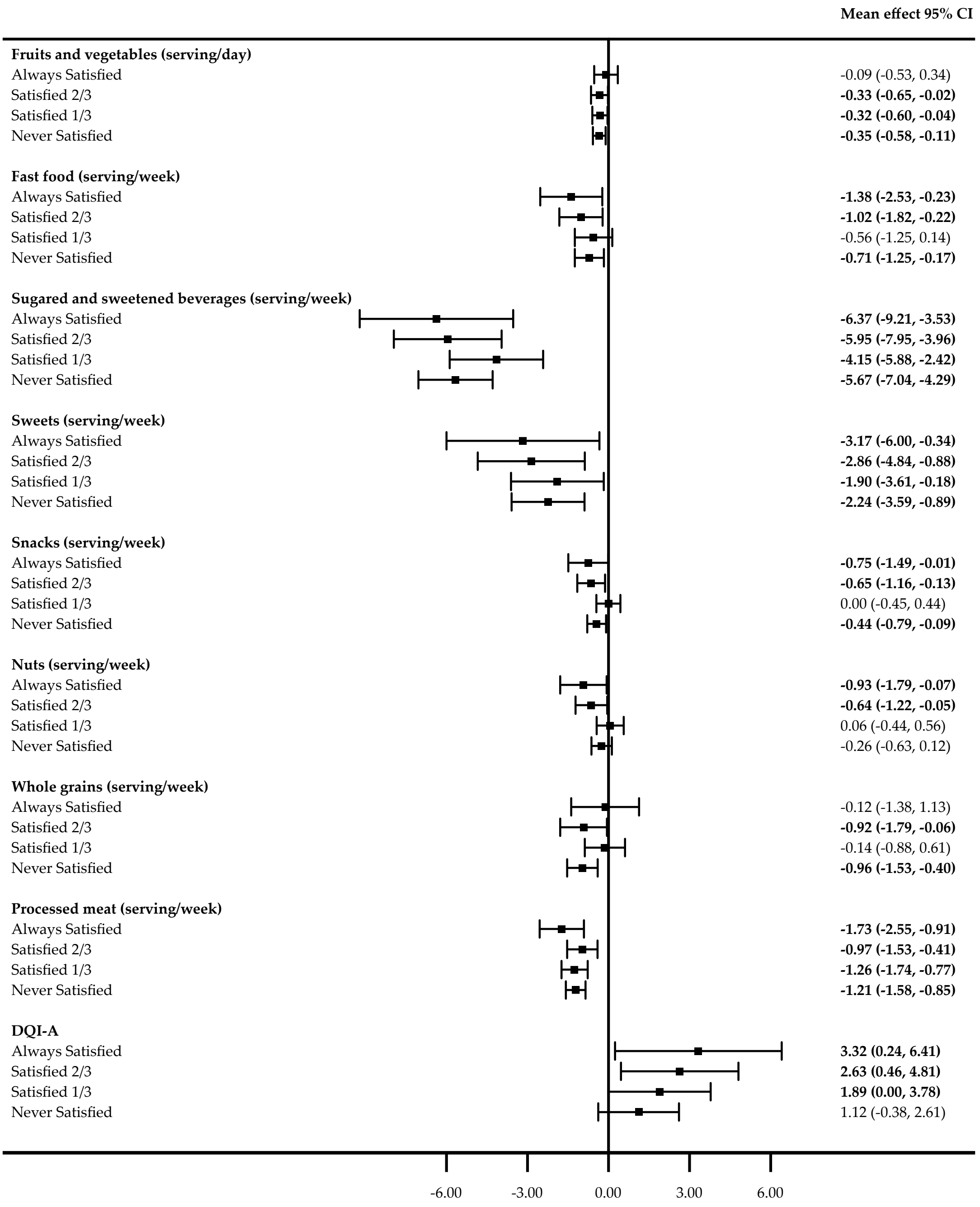
Mean effect 95% CI
Fruits and vegetables (serving/day)
Always Satisfied -0.09 (-0.53, 0.34)
Satisfied 2/3 -0.33 (-0.65, -0.02)
Satisfied 1/3 -0.32 (-0.60, -0.04)
Never Satisfied -0.35 (-0.58, -0.11)
Fast food (serving/week)
Always Satisfied -1.38 (-2.53, -0.23)
Satisfied 2/3 -1.02 (-1.82, -0.22)
Satisfied 1/3 -0.56 (-1.25, 0.14)
Never Satisfied -0.71 (-1.25, -0.17)
Sugared and sweetened beverages (serving/week)
Always Satisfied -6.37 (-9.21, -3.53)
Satisfied 2/3 -5.95 (-7.95, -3.96)
Satisfied 1/3 -4.15 (-5.88, -2.42)
Never Satisfied -5.67 (-7.04, -4.29)
Sweets (serving/week)
Always Satisfied -3.17 (-6.00, -0.34)
Satisfied 2/3 -2.86 (-4.84, -0.88)
Satisfied 1/3 -1.90 (-3.61, -0.18)
Never Satisfied -2.24 (-3.59, -0.89)
Snacks (serving/week)
Always Satisfied -0.75 (-1.49, -0.01)
Satisfied 2/3 -0.65 (-1.16, -0.13)
Satisfied 1/3 0.00 (-0.45, 0.44)
Never Satisfied -0.44 (-0.79, -0.09)
Nuts (serving/week)
Always Satisfied -0.93 (-1.79, -0.07)
Satisfied 2/3 -0.64 (-1.22, -0.05)
Satisfied 1/3 0.06 (-0.44, 0.56)
Never Satisfied -0.26 (-0.63, 0.12)
Whole grains (serving/week)
Always Satisfied -0.12 (-1.38, 1.13)
Satisfied 2/3 -0.92 (-1.79, -0.06)
Satisfied 1/3 -0.14 (-0.88, 0.61)
Never Satisfied -0.96 (-1.53, -0.40)
Processed meat (serving/week)
Always Satisfied -1.73 (-2.55, -0.91)
Satisfied 2/3 -0.97 (-1.53, -0.41)
Satisfied 1/3 -1.26 (-1.74, -0.77)
Never Satisfied -1.21 (-1.58, -0.85)
DQI-A
Always Satisfied 3.32 (0.24, 6.41)
Satisfied 2/3 2.63 (0.46, 4.81)
Satisfied 1/3 1.89 (0.00, 3.78)
Never Satisfied 1.12 (-0.38, 2.61)
-6.00
-3.00
0.00
3.00
6.00

b) Girls

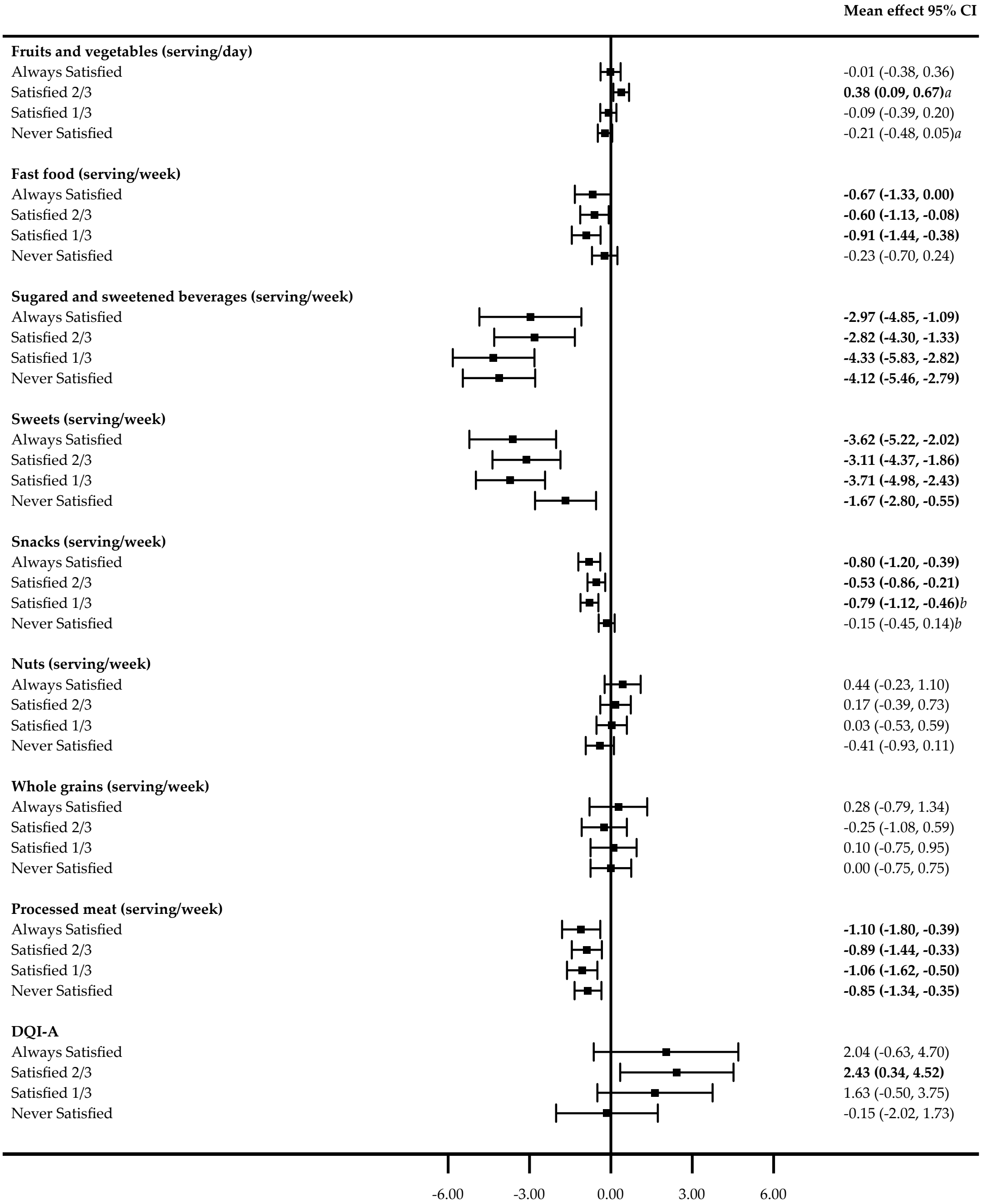

Supplemental Figure S2. Mean change estimates for food consumption from baseline to 4-year follow-up by BIS trajectory and gender.

BIS, body image satisfaction, DQI-A (ranged from -33/100%), diet quality index for adolescents. This analysis includes participants with data in the BIS variables in all three assessments. Estimated marginal means (95% confidence intervals (CI)) obtained from multilevel linear mixed-effects models. Fixed effects were BIS trajectory, age, nutritional status (underweight/normal weight/overweight/obesity), educational level (low/intermediate/high/unknown), migrant background (yes/no/unknown), randomization group (long term intervention/short term intervention/control), moderate-to-vigorous physical activity and sexual maturity status, and food consumption (servings/week) at baseline. Region (Madrid/Barcelona) and schools were handled as random effects. Significant differences (p≤0.05) are presented in bold. a, significant differences between satisfied 2/3 vs never satisfied; b, significant differences between satisfied 1/3 vs never satisfied .

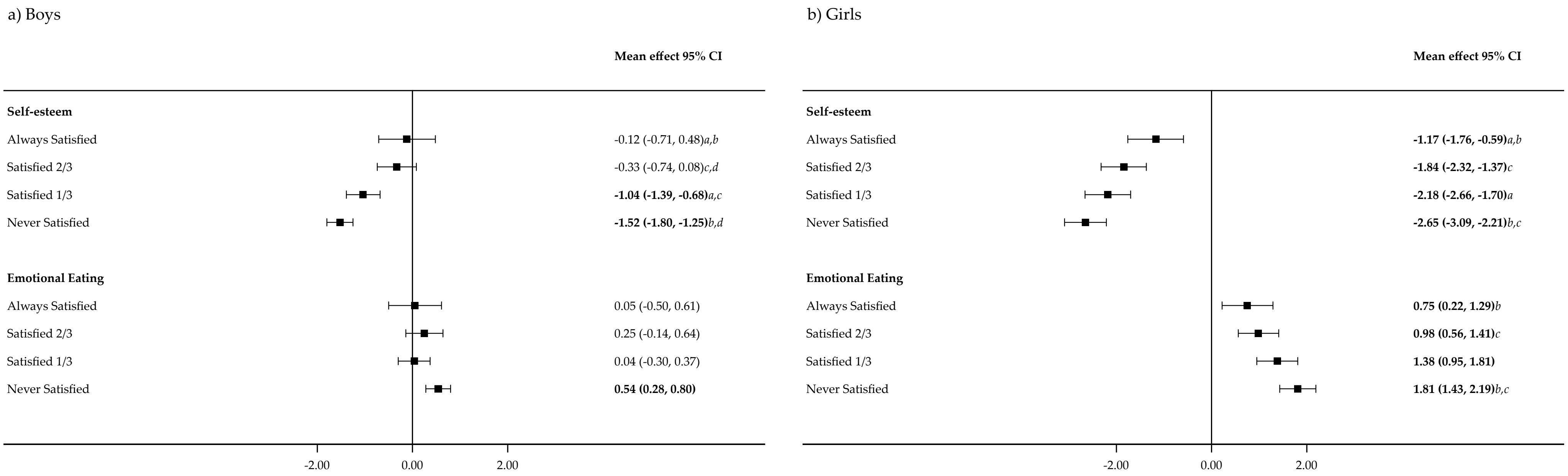

Supplemental Figure S3. Mean change estimates from baseline to 4-year follow-up for self-esteem and emotional eating by BIS trajectory and gender.

BIS, body image satisfaction. Emotional eating scale ranged 1-12 points, and self-esteem score ranged 1-20 points. This analysis includes participants with data in the BIS variables in all three assessments. Estimated marginal means (95% confidence intervals (CI)) were obtained from multilevel linear mixed-effects models. Fixed effects were BIS trajectory, age, nutritional status (underweight/normal weight/overweight/obesity), educational level (low/intermediate/high/unknown), migrant background (yes/no/unknown), randomization group (long term intervention/short term intervention/control), moderate-to-vigorous physical activity, sexual maturity status, and self-esteem or emotional eating score at baseline (continuous variable). Region (Madrid/Barcelona) and schools were handled as random effects. Significant differences (p≤0.05) are presented in bold. a, significant differences between always satisfied vs satisfied 1/3; b, always satisfied vs never satisfied; c, satisfied 1/3 vs satisfied 2/3; satisfied 2/3 vs never satisfied.
