# Supplementary figures and images for: Body Image Satisfaction, Food Consumption, Diet Quality, and Emotional Management in Adolescence: A Longitudinal Analysis from the SI! Program for Secondary Schools Trial
Source: Nutrients. 2025 Dec 12;17(24):3882. doi: 10.3390/nu17243882 (PMC12736312; doi:10.3390/nu17243882)

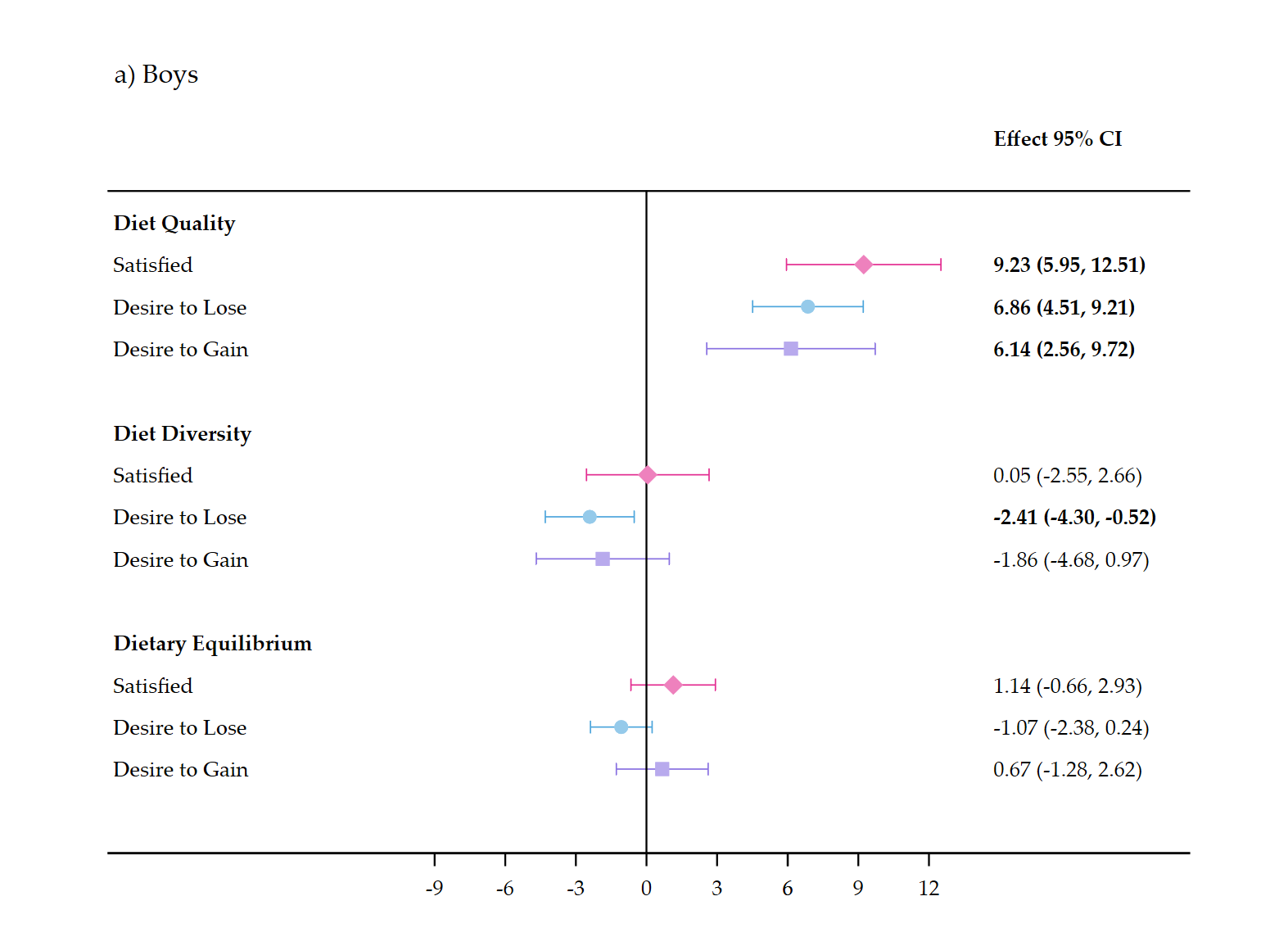

Supplement: Supplementary file 1 [file nutrients-17-03882-s001.zip › Supp Figure 1_DQI-A components_Boys.tif]

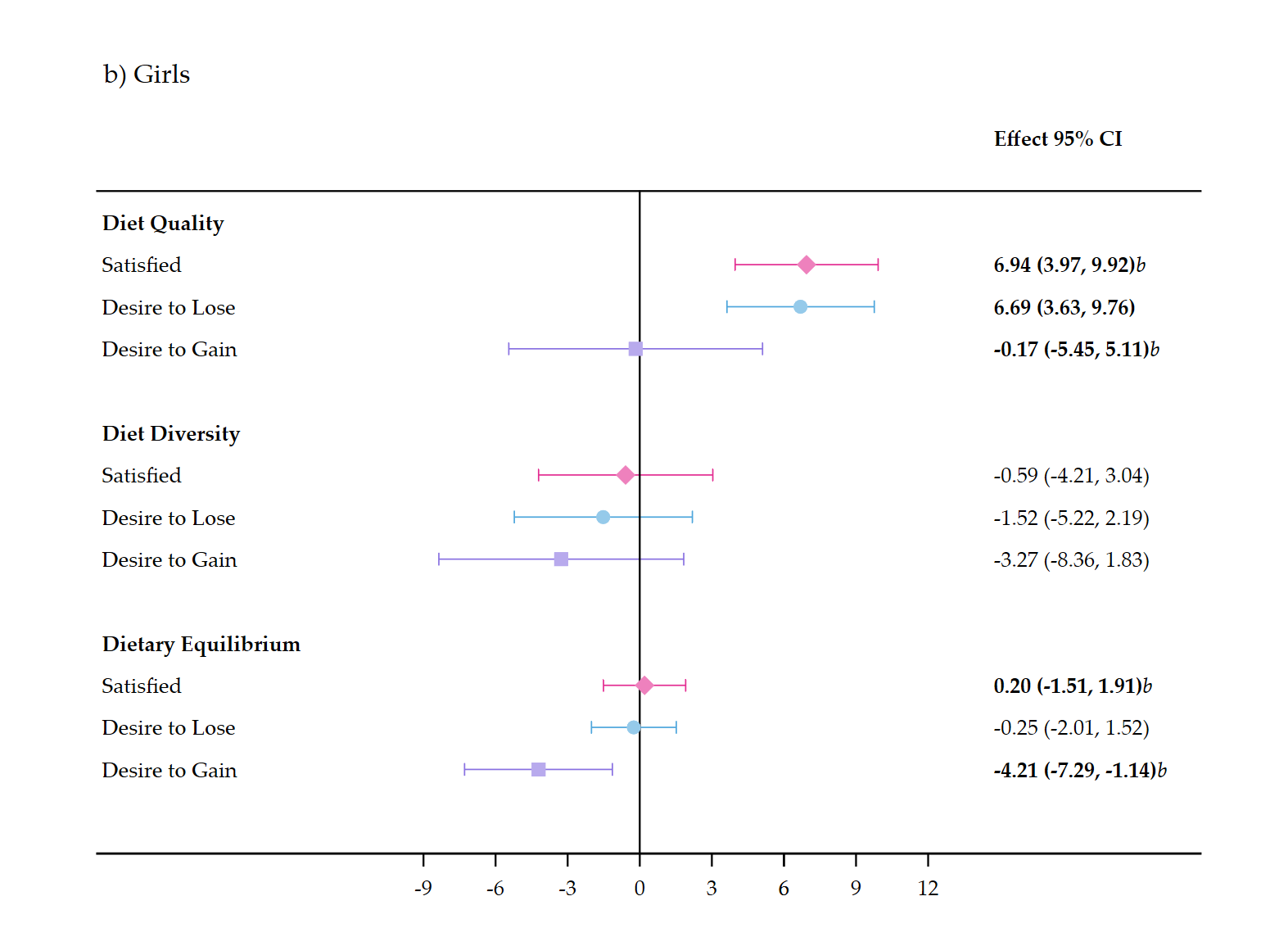

Supplement: Supplementary file 1 [file nutrients-17-03882-s001.zip › Supp Figure 1_DQI-A components_Girls.tif]

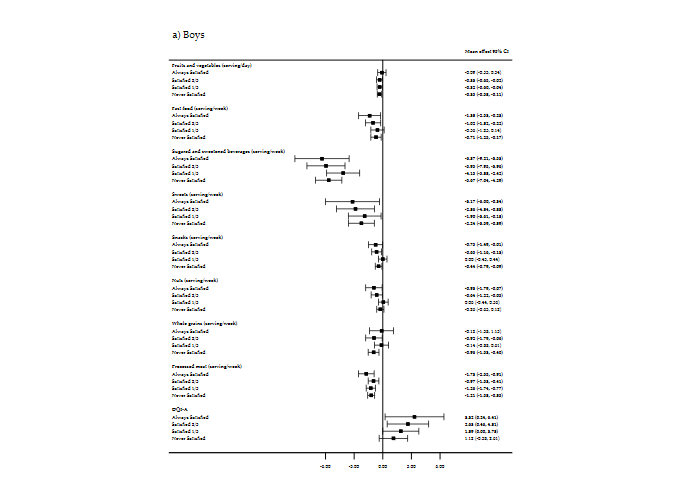

Supplement: Supplementary file 1 [file nutrients-17-03882-s001.zip › Supp Figure 2_BIS trajectories & Diet_Boys.tif]

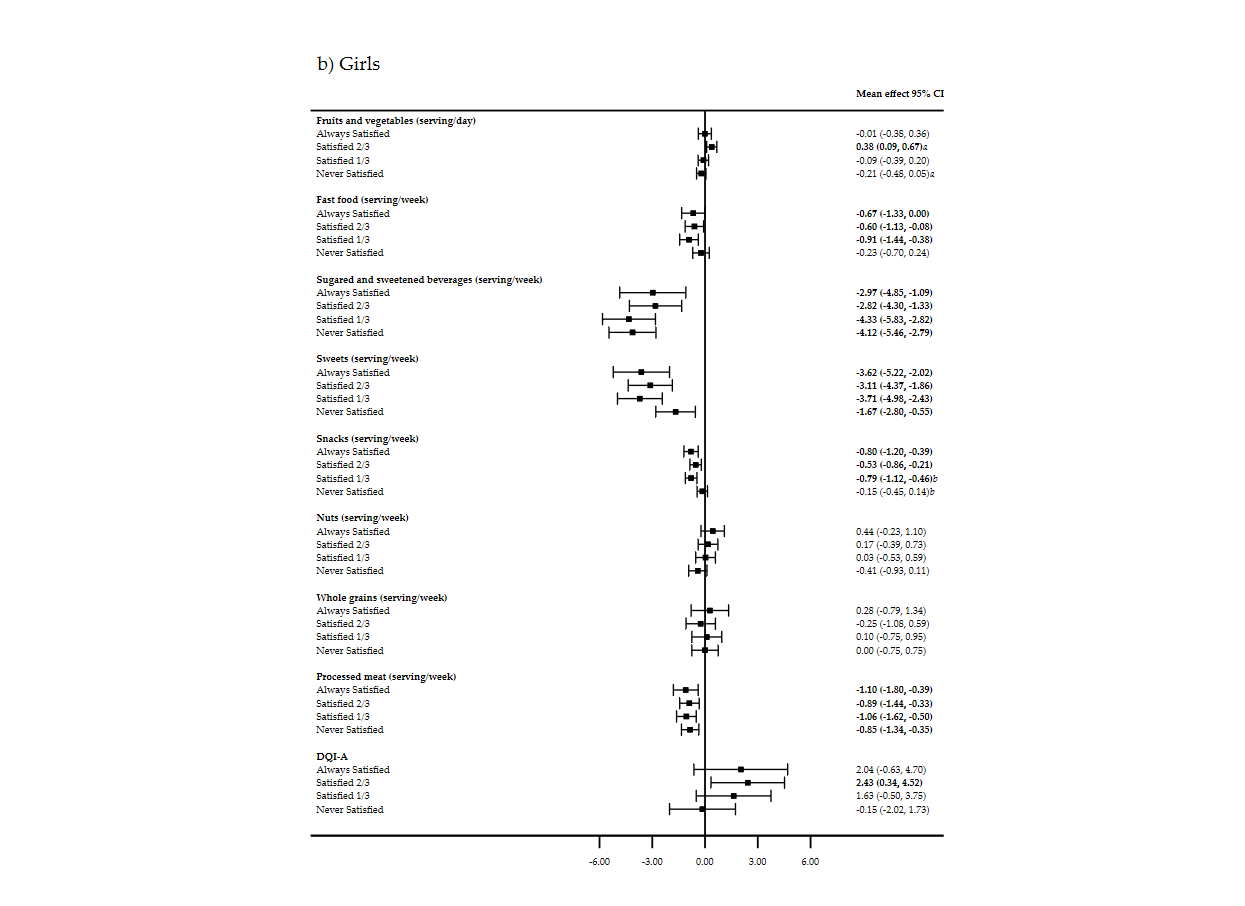

Supplement: Supplementary file 1 [file nutrients-17-03882-s001.zip › Supp Figure 2_BIS trajectories & Diet_Girls.tif]

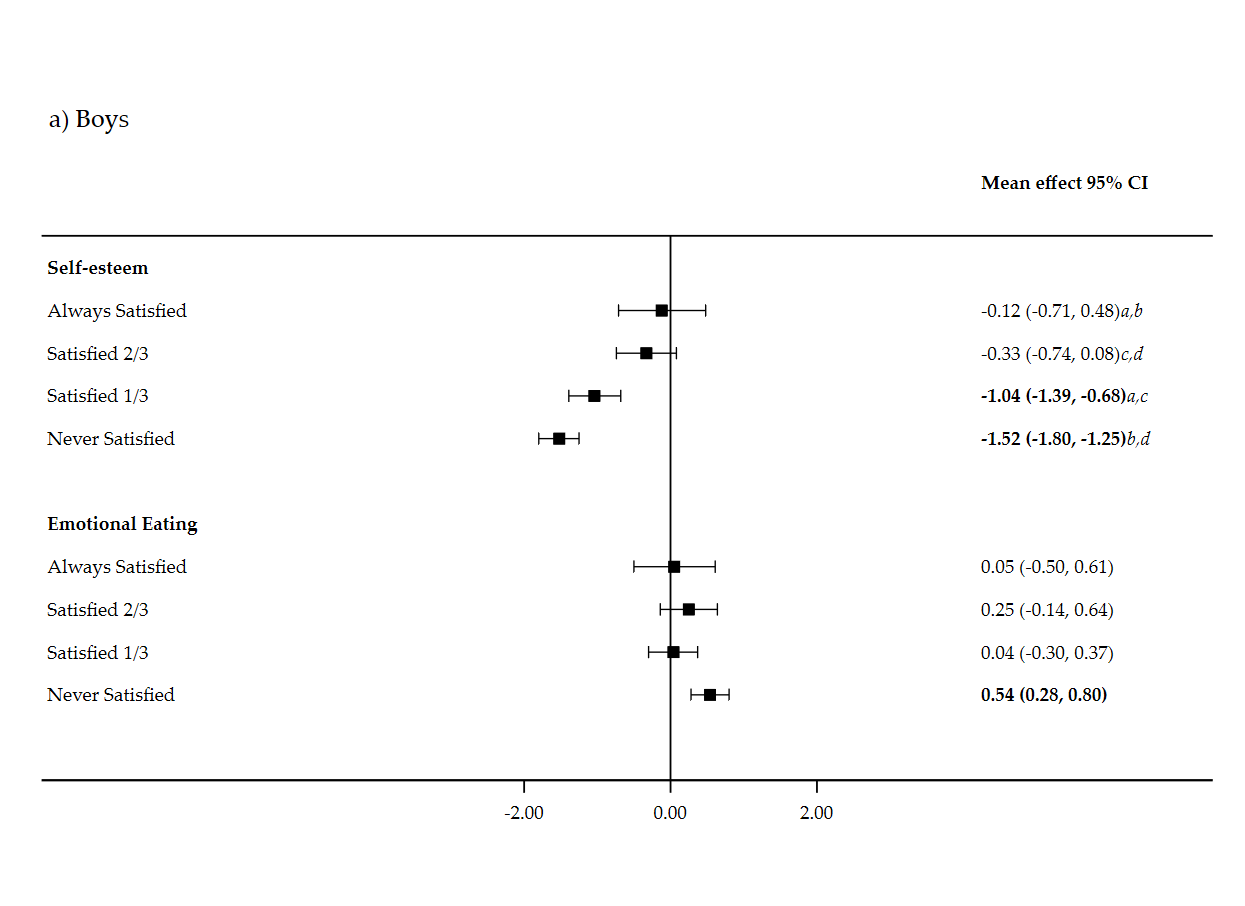

Supplement: Supplementary file 1 [file nutrients-17-03882-s001.zip › Supp Figure 3_BIS trajectories & Emotions_Boys.tif]

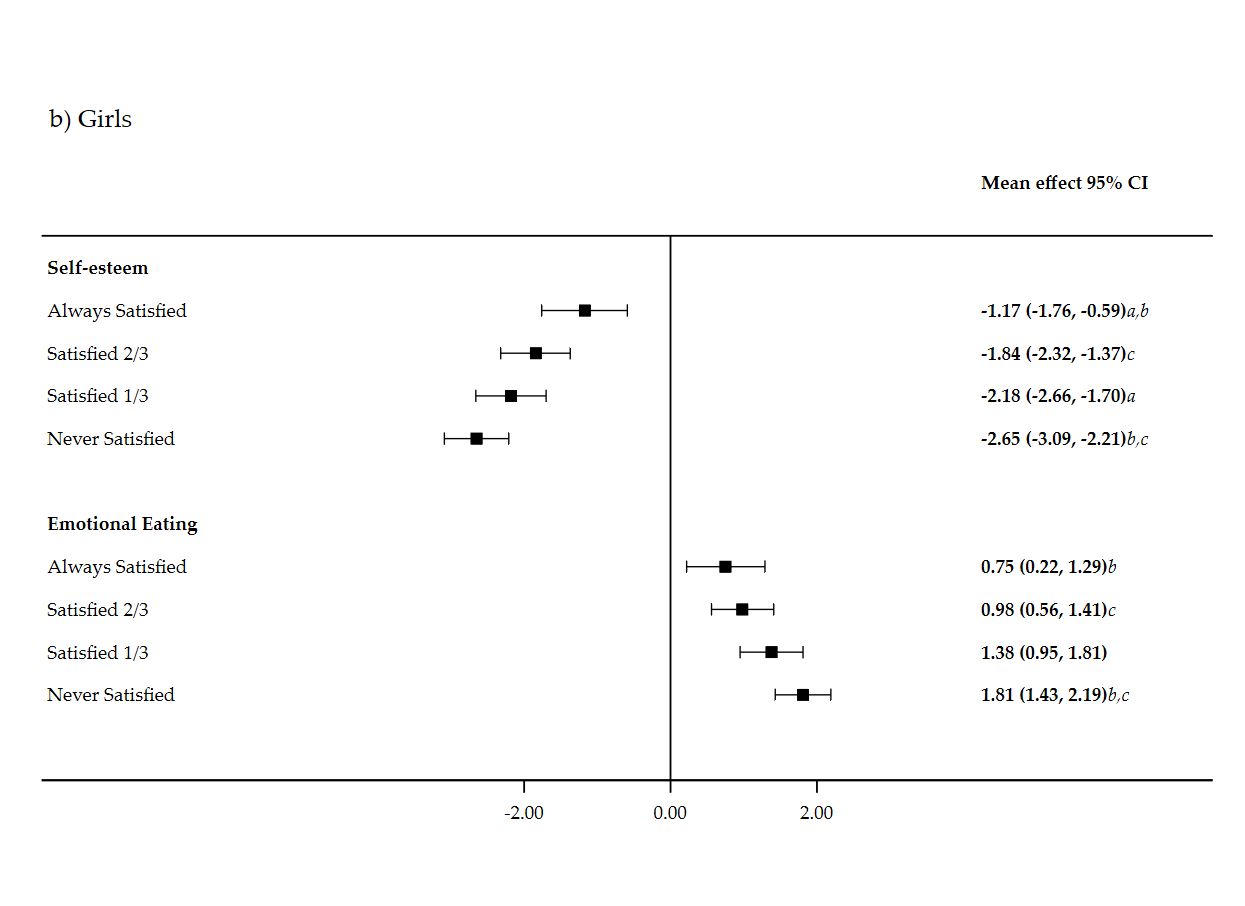

Supplement: Supplementary file 1 [file nutrients-17-03882-s001.zip › Supp Figure 3_BIS trajectories & Emotions_Girls.tif]
